# Isolation of a Novel Lytic *Pseudomonas aeruginosa* Phage Henu5 and Fitness Costs of Phage‐Driven Resistance

**DOI:** 10.1111/1751-7915.70389

**Published:** 2026-05-28

**Authors:** Salwa E. Gomaa, Jiawen Shen, Jingjing Li, Yangyang Liu, Chaonan Ma, Qiming Li, Guanbin Qi, Tieshan Teng

**Affiliations:** ^1^ Department of Pulmonary and Critical Care Medicine Huaihe Hospital of Henan University, Henan University Kaifeng Henan China; ^2^ Henan International Joint Laboratory for Nuclear Protein Regulation, School of Basic, Medical Sciences Henan University Kaifeng Henan China; ^3^ Department of Microbiology and Immunology Faculty of Pharmacy, Zagazig University Zagazig Egypt

**Keywords:** fitness costs, phage, *Pseudomonas aeruginosa*, resistance

## Abstract

Phage therapy is increasingly recognised as a potential approach against antibiotic‐resistant infections. Analogous to the rise of antibiotic resistance, bacteria can also evolve phage resistance, a process that frequently entails fitness costs. In this study, a new lytic *Pseudomonas aeruginosa* phage (Henu5) was isolated and characterised. Henu5 was classified within the *Caudoviricetes* class and displayed good biological characteristics. The Henu5 genome possesses 92,558 bp of linear dsDNA and a GC content of 49.36%. Additionally, three phage‐resistant mutants, R3, R6 and R14, were isolated and investigated for adaptive trade‐offs. Compared to wild‐type, the phage‐resistant mutants mostly exhibited fitness costs in growth, altered morphology, impaired adsorption, reduced biofilm and pyocyanin production and impaired motility, which was confirmed by transmission electron microscopy, as well as increased susceptibility to various antibiotics. In vivo experiments revealed improved survival, alongside diminished colonisation and pathogenicity. Comparative genomics identified mutations in the genes *pilQ* (R3 and R14) and *pilR* (R6), both crucial for type IV pili (T4P) biosynthesis and potentially conferring phage resistance, with additional mutations related to the obtained trade‐offs. Transcriptomic and real‐time quantitative PCR proved that T4P‐related genes, together with the *hmgA*, *galU*, *wzy*, *fliG*, *pslA*, *mexH* and *exoY* genes, were downregulated in all resistant mutants, while the *qscR* and *mvaT* genes were upregulated. Collectively, while phage resistance evolution remains an issue, this study suggests that the fitness costs of the phage‐resistant mutants might be invaluable for effective phage therapies.

## Graphical abstract

This study isolated and identified a novel lytic phage that infects *Pseudomonas aeruginosa* . Henu5 exhibited good physicochemical stability. Additionally, the phage‐resistant mutants showed fitness trade‐offs in growth, virulence and antibiotic susceptibility, along with in vivo fitness, which might direct future phage selection in clinical settings.

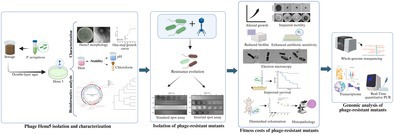

## 1 Introduction

*Pseudomonas aeruginosa* is a significant Gram‐negative pathogen that causes various community‐ and hospital‐acquired infections, including pneumonia, wound infections, urinary tract infections and bacteremia (Ugwuanyi et al. 2021). *Pseudomonas aeruginosa* pathogenicity is driven by a repertoire of virulence factors, among them pili, flagella, pyocyanin and biofilm formation (Zhang et al. 2025). It also exhibits intrinsic resistance to multiple classes of antibiotics, such as β‐lactams, aminoglycosides and fluoroquinolones, leading to high morbidity and mortality rates (Chegini et al. 2020). Based on the US Centers for Disease Control and Prevention, *Pseudomonas aeruginosa* is estimated to cause roughly 51,000 healthcare‐associated infections in the US each year, of which approximately 13% are multidrug‐resistant (MDR) and are responsible for about 400 deaths annually (CfD 2013). Additionally, *Pseudomonas aeruginosa* is categorised among the ‘high priority’ group of the WHO Bacterial Priority Pathogens List, posing a substantial burden in clinical anti‐infection treatment (Jesudason 2024). Carbapenem is currently used as the last‐resort antibiotic against *Pseudomonas aeruginosa* infections. However, its widespread use has led to the emergence of carbapenem‐resistant *Pseudomonas aeruginosa* , necessitating the urgent need for innovative therapeutics (Bubonja‐Sonje et al. 2015).

Phage therapy, viruses that kill pathogenic bacteria, is increasingly recognised as a promising approach to combat MDR infections (Abedon 2019). Earlier reports attested to the efficacy of phage therapy against *Pseudomonas aeruginosa* infections in experimental models and in clinical settings (Rubalskii et al. 2020; Chen et al. 2021; Nour El‐Din et al. 2025). Despite this strong evidence, one of the major obstacles toward phage therapy is that bacteria readily evolve phage resistance, which greatly impedes infection (Egido et al. 2022). A previous retrospective case series reported phage therapy failure in two patients with *Pseudomonas aeruginosa* infection despite in vitro susceptibility (Aslam et al. 2020). Phage resistance is mediated by diverse mechanisms, with adsorption inhibition being the most common (Kronheim et al. 2018). In Gram‐negative bacteria, phages typically interact with various surface receptors to initiate infection, including lipopolysaccharides (LPS), type IV pili (T4P), outer membrane proteins (OMPs) and exopolysaccharides (EPS) (Bertozzi Silva et al. 2016). Bacterial surface receptors are highly complex and changeable; thereby, bacteria can modify receptor structure and number or mask them within the extracellular matrix during biofilm growth, preventing phage adsorption (Majkowska‐Skrobek et al. 2021). For example, a multi‐base insertion in the *waaH* gene involved in LPS biosynthesis resulted in impaired phage adsorption in a *Shigella dysenteriae* mutant (Gomaa et al. 2025).

Nevertheless, bacterial receptors generally have key physiological roles in the cell; accordingly, such receptor‐associated mutations might come with a cost to bacterial fitness with respect to virulence, antibiotic susceptibility and survival (Mi et al. 2023). Previous studies demonstrated that the phage‐resistant *Pseudomonas aeruginosa* mutants resulting from alteration in T4P‐related genes have entailed adaptive trade‐offs, ultimately causing reduced bacterial motility and biofilm formation (Li et al. 2022). However, the natural genomic versatility of *Pseudomonas aeruginosa* and the emergence of various phenotypes still need to be explored, especially those shaped by co‐evolution between phages and their bacterial hosts (De Smet et al. 2017). In this context, a deeper understanding of how host bacteria thwart phage infection and the possible physiological outcomes caused by phage resistance is pivotal for the implementation of phage therapy in clinical settings.

In the present study, a new lytic *Pseudomonas aeruginosa* *phage* (Henu5) was isolated and characterised. Additionally, three phage‐resistant mutants, designated R3, R6 and R14, were isolated from the same strain. Furthermore, we conducted phenotypic and genotypic comparative analyses, along with in vivo infection models to assess bacterial colonisation, virulence and survival. Overall, this study elucidates phage resistance and the potential trade‐offs that could pave the way for a successful application of phages against *Pseudomonas aeruginosa* infections.

## 2 Materials and Methods

### 2.1 Isolation, Purification and Propagation of Phage Henu5

Phage Henu5 was isolated from the sewage sample collected from Kaifeng City, China, as previously described (Yehia et al. 2025). In brief, the filtered sewage sample was added to an equal volume of 2X Tryptone Soya Broth (TSB) containing an exponentially growing *Pseudomonas aeruginosa* (ATCC 9027) as a host. After overnight incubation at 37°C in a shaking incubator, the mixture was centrifuged, and the supernatant was screened for the existence of phage Henu5 using the spot assay. To purify phage Henu5, successive rounds of single plaque picking, resuspended in TSB, followed by the double‐layer agar (DLA) method, were performed until uniform plaque morphology was observed (Yehia et al. 2025). Phage Henu5 was propagated by picking a single plaque and inoculating it into 5 mL of TSB containing 50 μL of an exponentially growing *Pseudomonas aeruginosa* host strain. Following overnight incubation at 37°C with shaking, the resulting phage Henu5 lysate was harvested by centrifugation and filtration and kept at 4°C (Kumari et al. 2009).

### 2.2 Electron Microscopy of Phage Henu5

The morphology of the purified phage Henu5 was visualised under transmission electron microscopy (TEM). Twenty microliters of the concentrated phage Henu5 suspension were dripped onto a copper grid, followed by negative staining with 2% (*w*/*v*) phosphotungstic acid. The phage Henu5 particles were examined under a FEI Tecnai G2 Spirit 120 kV TEM (Tizro et al. 2018).

### 2.3 Optimal Multiplicity of Infection (MOI) and One‐Step Growth Curve

For optimal MOI determination, an exponentially growing *Pseudomonas aeruginosa* culture (10^8^ CFU/mL) was mixed with phage Henu5 at MOIs of 1 × 10^−4^–1 × 10 and incubated at 37°C for 4 h with shaking. Then, the samples were centrifuged, and phage Henu5 titre was assessed in the supernatants using the DLA method (Wang et al. 2022). A one‐step growth curve was employed to determine phage Henu5 latent period and the burst size. In brief, phage Henu5 was mixed with an exponentially growing *Pseudomonas aeruginosa* host strain at the optimal MOI and incubated for 15 min. Then, the mixture was centrifuged, the pellet was resuspended in fresh TSB, and re‐incubated. Samples were collected every 20 min for phage titre determination using the DLA technique (Gomaa et al. 2025).

### 2.4 Phage Henu5 Stability Testing

The influence of temperature, pH and chloroform on phage Henu5 lytic activity was investigated. Phage Henu5 was subjected to varying temperatures (4°C–100°C), pH ranges (2–12) and different ratios of chloroform (0%–80%) for 60 min. The residual phage Henu5 activity was assessed using the DLA method (Yehia et al. 2025).

### 2.5 Phage Genome Sequencing and Data Analysis

The genomic DNA of phage Henu5 was extracted as previously described (Lu et al. 2013). Sequencing was carried out using the Illumina NovaSeq 6000 sequencer (300 bp reads; Sangon Biotech Co. Ltd., Shanghai, China). Trimmomatic (v0.36) (Brown et al. 2017) was used for raw reads trimming, SPAdes (v3.15) (Bankevich et al. 2012) was employed for assembly of the trimmed reads, and Gapcloser (v1.11) (Boetzer and Pirovano 2012) was used for filling gaps. The open reading frames (ORFs) prediction and functional annotation were performed using the ORF finder tool (https://www.ncbi.nlm.nih.gov/orffinder/). The phage circular genomic map was created using Proksee (https://proksee.ca). To identify tRNAs, tRNAscan‐SE (http://lowelab.ucsc.edu/tRNAscan‐SE/) was utilised. The presence of the antibiotic resistance genes was screened using the Comprehensive Antibiotic Resistance Database (CARD) (https://card.mcmaster.ca/analyze/rgi), while virulence determinants and integrases were predicted using the Virulence Factors Database (VFDB) (https://www.mgc.ac.cn/VFs/) and PhageAI (https://www.phage.ai/), respectively.

Taxonomic assignment was conducted using the taxMyPhage tool (https://ptax.ku.dk/). The phylogenetic analysis of whole‐genome sequences of phage Henu5 and closely related *Pseudomonas* phages was constructed using BLASTn analysis using the neighbour‐joining method in the MEGA 11 program with 1000 bootstrap repeats (Tamura et al. 2021). The Virus Intergenomic Distance Calculator (VIRIDIC) was used to calculate the intergenomic similarities between phage Henu5 and related phages (Moraru et al. 2020). The genome comparison between phage Henu5 and similar phages was performed using the Easyfig software (Sullivan et al. 2011).

### 2.6 Isolation of Phage‐Resistant Mutants

The phage‐resistant mutants were isolated as previously described (D'Andrea et al. 2020). In brief, an exponential phase *Pseudomonas aeruginosa* culture was infected with 10^10^ PFU/mL of phage Henu5. Then, the phage‐bacteria co‐culture was incubated overnight at 37°C with shaking before being plated on TSA plates. Afterwards, several single colonies were selected and subjected to three rounds of purification on TSA plates. The purified phage‐resistant mutants were kept in 20% glycerol at −80°C for further experiments. To verify their phage resistance, both standard spot and inverted spot assays were conducted (Zhao et al. 2024). The mutation frequency of the phage‐resistant mutants was calculated as previously described (Lopes et al. 2018).

### 2.7 Lysis Curve Assay

The lysis curve for the phage Henu5 was conducted by mixing the phage (MOI of 1) with the exponentially grown *Pseudomonas aeruginosa* cultures of either the wild‐type (WT) or the phage‐resistant mutant (R3, R6 and R14) strains. Subsequently, the samples were incubated at 37°C for 3 h in a shaking incubator, with the turbidity measured at OD_600 nm_ every 30 min. As controls, bacterial cultures without phage addition were used (García‐Cruz et al. 2024).

### 2.8 Adsorption Assay

The phage adsorption assay was performed as previously described (Gomaa et al. 2025). Briefly, phage Henu5 was mixed with exponentially growing cultures of the WT, R3, R6 and R14 strains at the optimal MOI and incubated at 37°C for 15 min, with aliquots (1 mL) collected at 3‐min intervals and subjected to centrifugation. Subsequently, the free phage titers were assessed in the supernatants using the DLA method.

### 2.9 Motility Assays

Twitching motility and swarming motility were assayed as previously described with some modifications (Rashid and Kornberg 2000). For the twitching assay, Muller Hinton Agar (MHA) plates (1% Bacto agar) were stab‐inoculated with fresh colonies of the WT, R3, R6 and R14 strains. For the swarming assay, 5 μL aliquots of freshly prepared bacterial suspensions (OD_600 nm_ of 0.08) were spotted onto MHA plates (0.5% Bacto agar). All plates were incubated at 37°C for 48 h. The twitching zones were visualised after agar removal by staining the plates with 1% crystal violet for 10 min. Then, the twitching and swarming plates were photographed, and the diameters of the motility zones were measured.

### 2.10 Electron Microscopy of *Pseudomonas aeruginosa* Strains

To visualise bacterial cells, TEM was employed (Jain et al. 2012). In brief, exponentially growing cultures of the WT, R3, R6 and R14 strains were dripped onto copper grids, negatively stained with 1% uranyl acetate. The grids were examined under a FEI Tecnai G2 Spirit 120 kV TEM.

### 2.11 Biofilm Formation Assay

Biofilm formation was investigated using 96‐well microtiter plates. In brief, overnight cultures of the WT, R3, R6 and R14 strains were adjusted to ~10^6^ CFU/mL in fresh TSB and dispensed into microtiter plates (200 μL/well). The plates were grown overnight at 37°C, and the biofilm formation was evaluated using a crystal violet assay. The absorbance was measured at 570 nm (Stepanović et al. 2007).

### 2.12 Pyocyanin Assay

The pyocyanin production was assessed as previously described (Cai et al. 2015; Fila et al. 2018). Briefly, 7.5 mL aliquots of cell‐free supernatants of the WT, R3, R6 and R14 strains were mixed with 4.5 mL aliquots of chloroform, followed by vortexing until a greenish‐blue colour was obtained. Subsequently, the mixtures were subjected to centrifugation, and 3 mL aliquots of the obtained blue pigment were transferred into a new tube, and 1.5 mL aliquots of 0.2 M HCl were added and shaken vigorously until a pink colour was achieved. The pyocyanin content was measured in the pink layer at OD_520 nm_, and the values were normalised to the corresponding culture densities measured at OD_600 nm_.

### 2.13 Antibiotic Susceptibility Assay

Minimal inhibitory concentrations (MICs) of cefepime (FEP), aztreonam (ATM), gentamicin (GEN), tobramycin (TOB), amikacin (AMK), ciprofloxacin (CIP) and levofloxacin (LEV) were determined against the WT, R3, R6 and R14 strains using the microdilution broth method following CLSI guidelines (CLSI 2021).

### 2.14 *Galleria mellonella* Infection Model

To assess the virulence of the WT, R3, R6 and R14 strains, the *G. mellonella* larvae infection model was used (Axline et al. 2025). In brief, exponentially growing bacterial cultures were pelleted, washed and resuspended in phosphate‐buffered saline (PBS). Larvae of 200–350 mg (Keyun Biotechnology, China) were randomly distributed into groups (10 larvae/group). Larvae of each group were injected with one bacterial suspension (~10^3^ CFU) at the final proleg site (10 μL/larva). PBS‐injected larvae were included as a control. Injected larvae were incubated in plastic Petri dishes in the dark at 37°C, and survival was monitored daily for 5 days. Larval death was inferred by the failure of movement in response to repeated agitations. The Kaplan–Meier survival curve was analysed using the Log‐rank test.

### 2.15 Mouse Infection Model

The colonisation potential of the WT, R3, R6 and R14 strains was investigated using a mouse infection model (Gordillo Altamirano et al. 2021). Briefly, female BALB/c mice (6 weeks old, 20–25 g weight) were purchased from Henan Skobes Biotechnology Co. Ltd. Mice were randomly allocated to six groups (6 mice each). The tested strains were adjusted to 10^8^ CFU in PBS, and each mouse group was intraperitoneally challenged with one strain (100 μL/mouse). PBS‐injected and non‐infected mice groups were included as controls. Twelve hours post‐infection, five mice per group were sacrificed, and the liver, lungs and kidneys were dissected, weighed and homogenised in PBS for bacterial load determination via CFU counts. In addition, at the same time point, tissue organs from one mouse were retrieved and fixed in buffered formalin (10%) for histopathological examination. The animal experiment was approved by Henan University's Subcommittee on Biomedical Research Ethics (Approval number: HUSOM2025‐765).

### 2.16 Comparative Genomic Analysis

For whole genome resequencing of the WT, R3, R6 and R14 strains, the Coolaber Bacterial DNA Extraction Kit (Coolaber, Beijing, China) was used for DNA extraction following the manufacturer's protocol. The libraries generation and sequencing were conducted by Qingke Biotechnology Co. Ltd. (Beijing, China) using the Illumina NovaSeq X Plus (Illumina, San Diego, USA) sequencer with the 2 × 150 bp paired‐end sequence kit following the manufacturer's guidelines. For comparative genome sequencing, *Pseudomonas aeruginosa* PAO1 (GenBank GCA_000006765.1) was used as a reference strain.

### 2.17 Transcriptome Analysis

For RNA sequencing, the total bacterial RNA was extracted using the Coolaber Bacterial RNA Extraction Kit (Coolaber, Beijing, China). Transcriptome sequencing was performed by Tsingke Biotechnology Co. Ltd. (Beijing, China) using the NovaSeq X Plus platform. Data analysis started with quality assessment and control of the raw sequencing data using fastp (https://github.com/OpenGene/fastp) (Chen et al. 2018). This was followed by an evaluation of rRNA contamination by aligning reads to the Rfam database using BLAST. The resulting high‐quality clean data were then aligned to the reference genome using HiSat2/TopHat2 (https://www.ccb.jhu.edu/software/hisat2/index.shtml, https://www.ccb.jhu.edu/software/tophat/index.shtml) (Kim et al. 2013; Kim et al. 2019). Differential expression analysis was conducted using DESeq2 (for experiments with biological replicates) (Anders and Huber 2010), (https://www.rdocumentation.org/packages/DEGseq/versions/1.26.0) or edgeR (for experiments without biological replicates), (https://www.bioconductor.org/packages/2.12/bioc/html/edgeR.html), based on read counts mapped to genes, employing a negative binomial distribution model. The default criteria for identifying significantly differentially expressed genes (DEGs) were an FDR < 0.05 and an absolute log2 fold change (|log2 FC|) ≥ 1. Based on the screened DEGs and their enriched pathways, volcano plots, Gene Ontology (GO) enrichment analysis plots and Kyoto Encyclopedia of Genes and Genomes (KEGG) pathway enrichment analysis plots were generated using the ggplot2 package in R (version 4.3.1).

### 2.18 Real‐Time Quantitative PCR (RT‐qPCR) Analysis

To validate RNA sequencing, the relative expression levels of 17 DEGs were analysed using RT‐qPCR. In brief, suspensions of the WT, R3, R6 and R14 strains were grown to the exponential phase. The total RNA was extracted, and cDNA was synthesised in accordance with the manufacturer's protocol. RT‐qPCR was performed as previously described (Gomaa et al. 2025). The relative expression level of each gene was normalised to *rho* using the 2^−ΔΔCt^ method. The primer sequences are shown in Table S1.

### 2.19 Statistical Analysis

Statistical analyses were carried out using GraphPad Prism v.10.1. Unless otherwise mentioned, Student's *t*‐test was employed for analysis. The data was expressed as the mean ± standard error of the mean (SEM) from three independent experiments.

## 3 Results

### 3.1 Isolation and Characterisation of Phage Henu5

**FIGURE 1:**
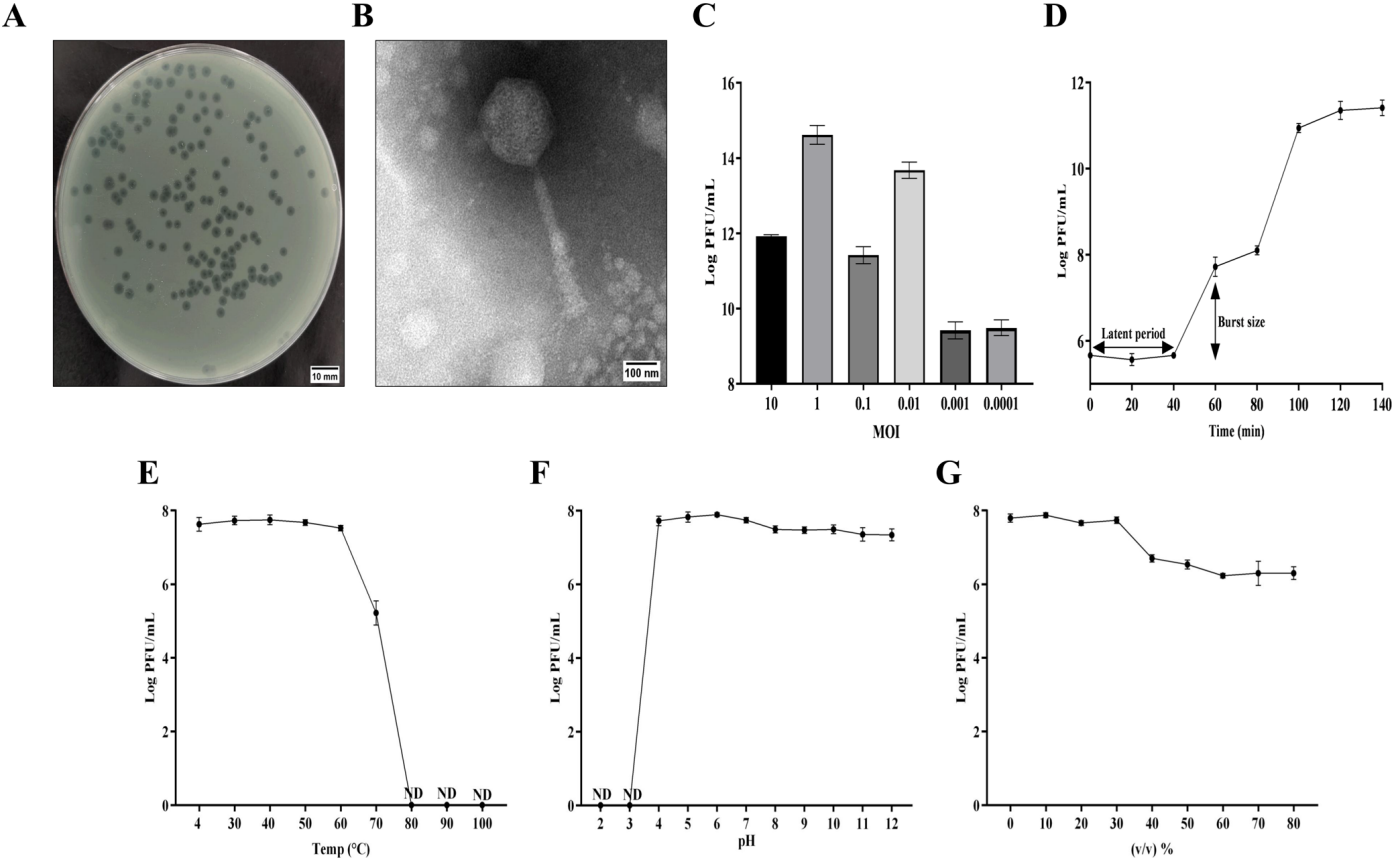
Morphological and biological characterisation of Henu5. (A) Henu5 plaques formed on *P. aeruginosa* lawn. Scale bar, 10 mm. (B) TEM micrograph of Henu5 particles. Scale bar, 100 nm. (C) The optimal MOI of Henu5. (D) The one‐step growth curve of Henu5. (E–G) Stability analysis of Henu5 after 1 h of incubation. (E) Thermal stability. (F) pH stability. (G) Chloroform stability. ND, not detected.

Phage Henu5 was isolated from sewage using *Pseudomonas aeruginosa* (ATCC 9027) as the host strain. Phage Henu5 produced opaque halo zones surrounding clear plaques on DLA plates after 24 h (Figure 1A). Under TEM, phage Henu5 particles had an icosahedral head, a long contractile tail, and a baseplate with fibres (Figure 1B). According to the updated ICTV guidelines, phage Henu5 is classified within the *Caudoviricetes* class, which represents a higher‐level classification compared to the traditional family/genus‐based phage taxonomy.

**FIGURE 2:**
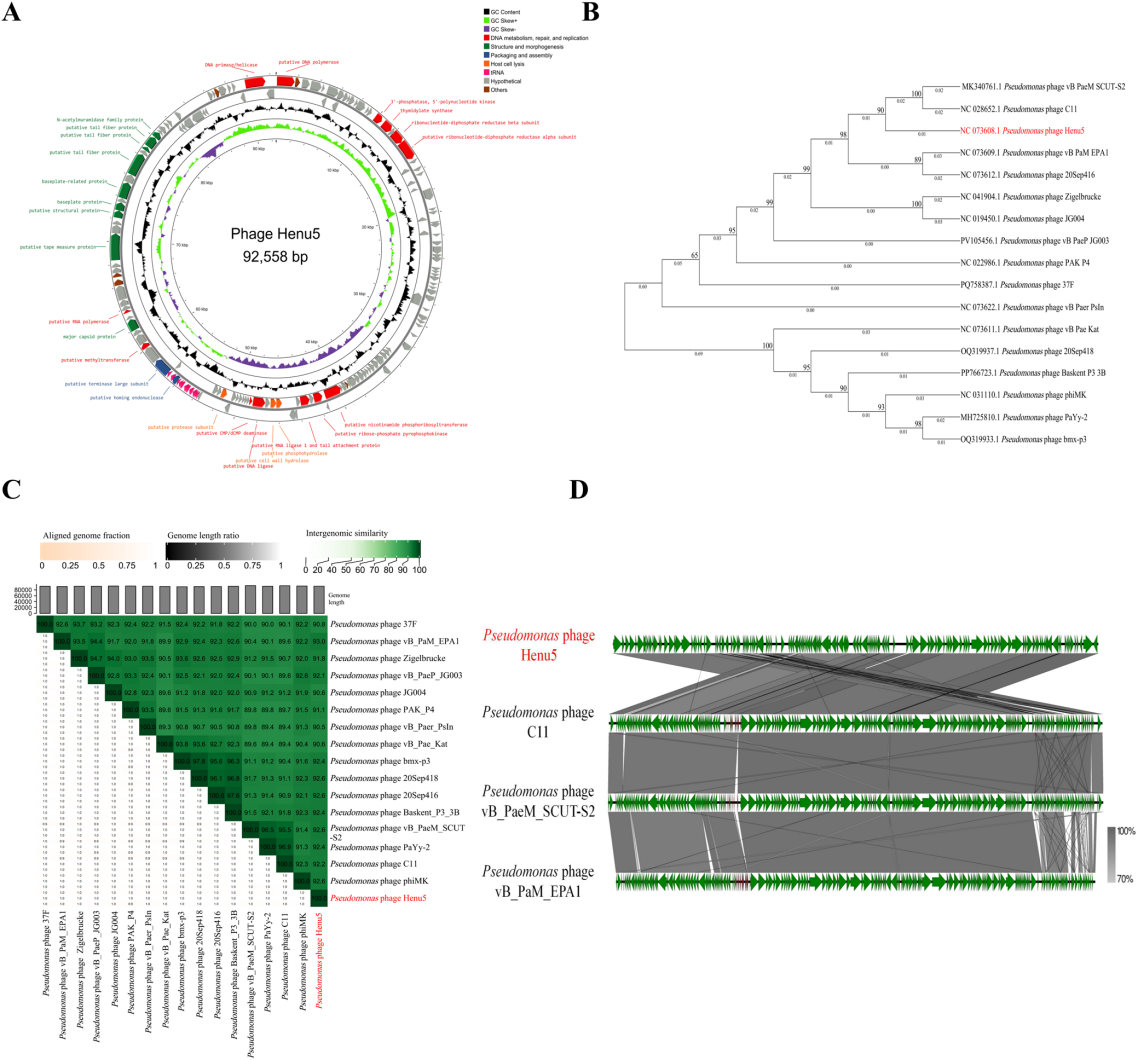
Genomic characterisation of Henu5. (A) The genomic circular map of Henu5 created using Proksee (https://proksee.ca). The inner rings represent genome location, GC skew+ (light green), GC skew− (purple) and GC content (black). Coloured arrows represent ORFs. Functional ORFs are classified according to functional assignments into four modules: DNA metabolism, repair and replication (red); structure (dark green); packaging and assembly (blue); and host cell lysis (orange). Hypothetical proteins are highlighted in grey. The tRNAs are indicated in pink. (B) Phylogenetic tree generated by MEGA 11 software using the complete sequence of Henu5 and 16 other closely related *Pseudomonas* phages. (C) VIRIDIC heatmap displaying the intergenomic similarities between Henu5 and related phages, constructed using VIRIDIC software. (D) Comparative genomic analysis between Henu5 and related sequences (C11, vB_PaeM_SCUT‐S2 and vB_PaM_EPA1) was performed by Easyfig. Green arrows indicate ORFs, and the shaded grey colour represents regions of sequence identity. Henu5 was coloured red.

Next, phage titers were evaluated at various MOIs using the DLA method. Phage Henu5 reached the highest titre of 5.47 × 10^14^ PFU/mL at an MOI of 1, suggesting this was the optimal MOI (Figure 1C). According to the one‐step growth curve, phage Henu5 had a latency of 40 min with a burst size of roughly 662 PFU/infected cell (Figure 1D). Thermal stability tests revealed that phage Henu5 tolerated temperatures up to 60°C. However, the phage activity reduced significantly at 70°C, with complete loss of activity at higher temperatures (Figure 1E). In addition, phage Henu5 retained its infectivity over wide pH values (4–12), while it could not survive at pH 2 and 3 (Figure 1F). Moreover, phage Henu5 titre decreased significantly upon exposure to chloroform concentration (> 30%); nevertheless, complete inactivation was not recorded (Figure 1G). Consequently, these characteristics support the potential suitability for developing phage Henu5 into clinically viable formulations that possess a good shelf‐life. To investigate genomic organisation, taxonomic classification and evolutionary relatedness of phage Henu5, genome sequencing followed by bioinformatic analysis was conducted. The genome sequence of phage Henu5 has been deposited in the GenBank database under accession number NC_073608.1. The phage Henu5 possesses a linear dsDNA genome of 92,558 bp with a GC content of 49.36%. Genome annotation revealed 145 open reading frames (ORFs) in phage Henu5 genome; 93 ORFs are located on the forward strand; and 52 ORFs are on the reverse strand. Among the identified ORFs, 32 ORFs constitute functional proteins, while 113 ORFs were annotated as hypothetical proteins. The functional proteins were classified into four categories: DNA metabolism, repair and replication; structure proteins; packaging and assembly proteins; and host cell lysis proteins (Figure 2A and Table S2). The online tRNAscan‐SE tool revealed the presence of 17 tRNAs in phage Henu5 genome (Table S3). Additionally, phage Henu5 genome did not contain integrases, antibiotic resistance genes or virulence genes, suggesting a virulent life cycle and further indicating that it is a suitable candidate for phage therapy.

Phage Henu5 was taxonomically classified as a member of the genus *Pakpunavirus* of the subfamily *Skurskavirinae* under the family *Vandenendeviridae* within the class *Caudoviricetes*. Whole‐genome phylogeny showed that phage Henu5 was closely related to phages C11 and vB_PaeM_SCUT‐S2, with percent identities of 94.74% and 97.52%, respectively (Figure 2B). VIRIDIC heatmap demonstrated that phage Henu5 shared the highest intergenomic similarity (93%) with vB_PaM_EPA1 phage (Figure 2C). Comparative genomic analysis through Easyfig revealed high homology among phages Henu5, C11, vB_PaeM_SCUT‐S2 and vB_PaM_EPA1, with gene similarity ranging from 70 to 100% (Figure 2D).

### 3.2 Isolation and Fitness Costs of Phage‐Resistant Mutants

**FIGURE 3:**
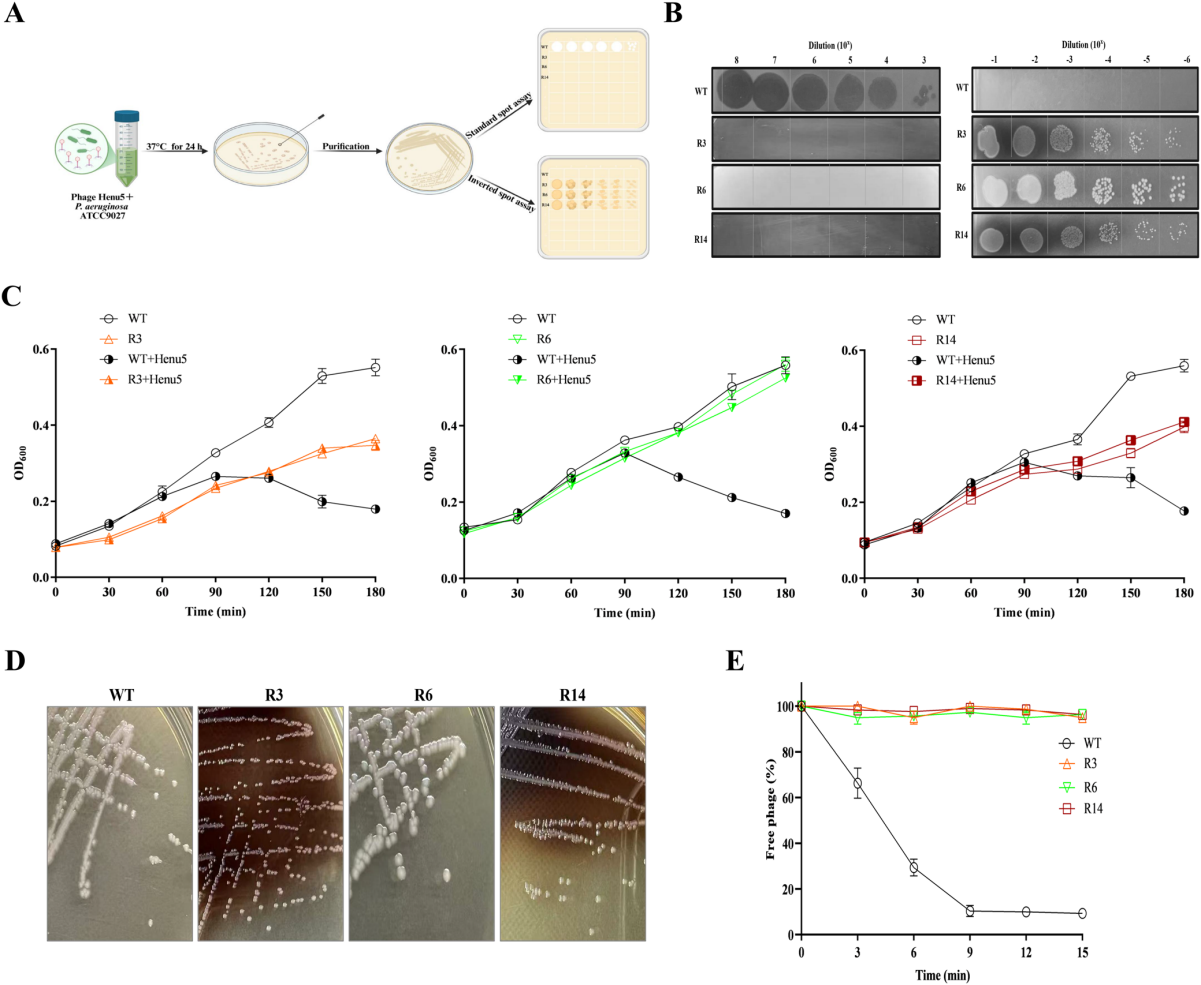
Characterisation of phage resistance. (A) Schematic of the isolation and verification of phage resistance. (B) The spot assays (left panel) and the inverted spot assays (right panel) of the wild‐type (WT) and the phage‐resistant mutants (R3, R6 and R14). (C) Growth kinetics of the WT strain and its derived mutants in the presence and absence of Henu5. The optical density (OD_600 nm_) was measured every 30 min over 3 h. (D) Colony morphology of the phage‐resistant mutants compared with the WT strain. (E) Adsorption of Henu5 onto the WT strain or its mutants. ImageJ was used to improve image quality for better visualisation.

The emergence of phage resistance in *Pseudomonas aeruginosa* was detected following a 24‐h co‐culture with its phage (Figure 3A). Two different phenotypes of the phage‐resistant mutants, white and brown, were identified based on colony pigmentation. One white mutant (R6) and two brown mutants (R3 and R14) were selected and confirmed for their phage resistance. The standard spot assay demonstrated that the R3, R6 and R14 strains were resistant to lysis by phage Henu5, as evidenced by the absence of plaque formation, contrary to the wild‐type (WT) strain. Additionally, on the inverted spot agar plates containing phage Henu5, the WT strain was unable to grow, while the R3, R6 and R14 strains experienced regular growth, suggesting the phage‐resistant phenotypes (Figure 3B). The white and brown mutants were detected at frequencies of ∼2.2 × 10^−8^ and 8 × 10^−9^, respectively.

In terms of growth characteristics, the phage‐resistant mutants displayed altered morphology on TSA plates compared to the WT strain. The R3 and R14 strains formed smaller colonies with brown pigment, whereas the R6 strain appeared to have slightly larger, white colonies. The results of the growth curve demonstrated that the growth of the WT strain was suppressed by phage Henu5, unlike the R3, R6 and R14 strains. Notably, the R3 and R14 exhibited slower growth rates than the WT strain, whereas no significant growth differences between the R6 and the WT strains were observed over the tested time intervals (Figure 3C,D).

**FIGURE 4:**
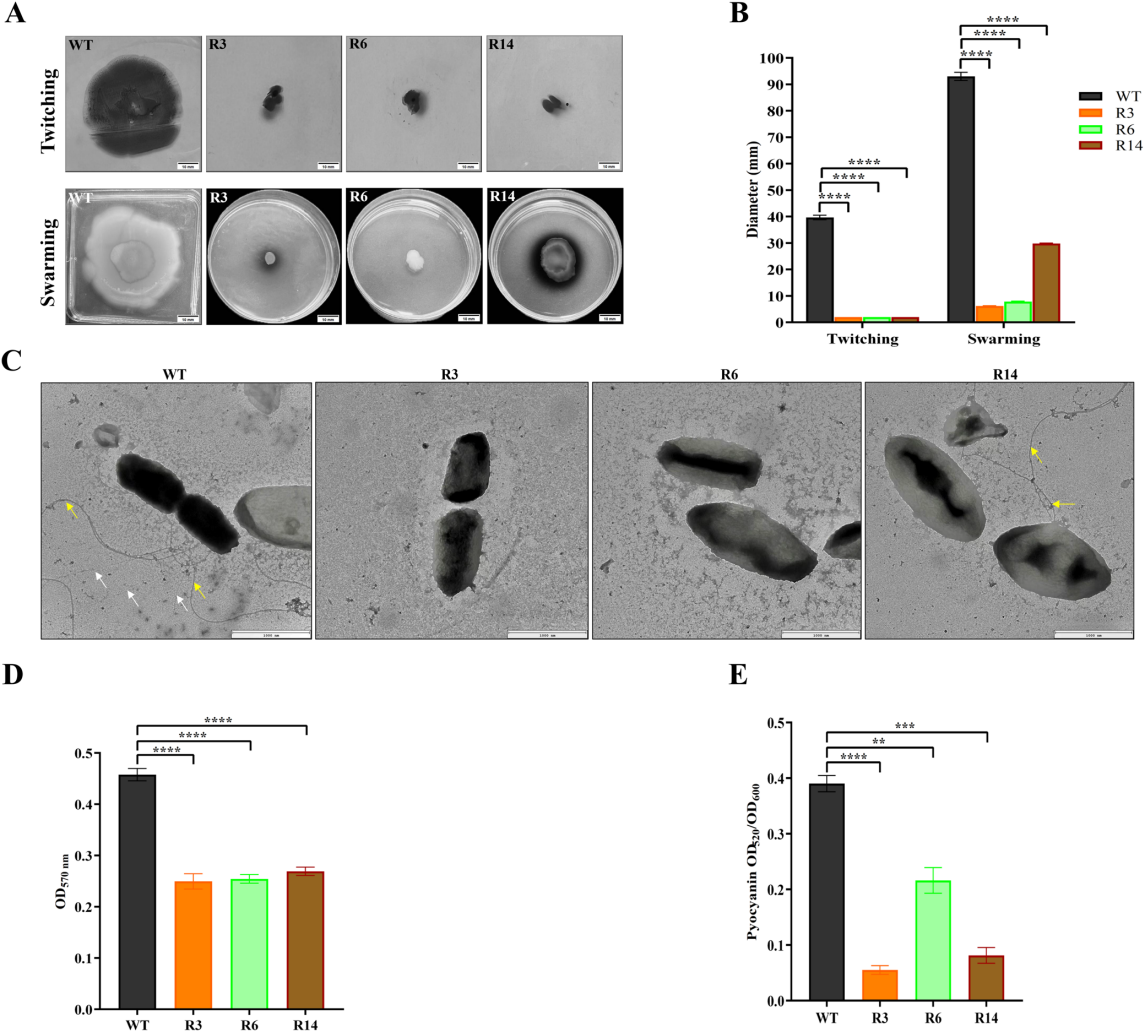
Virulence of the phage‐resistant mutants (R3, R6 and R14), relative to the wild‐type (WT) strain. (A) Representative photographs of twitching motility (upper panel) and swarming motility (lower panel) of *P. aeruginosa* strains after 48 h. Scale bar, 10 mm. (B) Quantification of twitching and swarming distances (mm) of *P. aeruginosa* strains. (C) TEM visualisation of *P. aeruginosa* strains. Yellow arrows point to the flagellum, and white arrows indicate type IV pili. The absence of arrows represents the lack of these appendages. Scale bar, 1000 nm. (D) Biofilm formation of *P. aeruginosa* strains after 24 h. The absorbance was measured at 570 nm (E) Pyocyanin production between *P. aeruginosa* strains. The pyocyanin measurements at OD_520 nm_ were normalised to the cell growth measured at OD_600 nm_. **p < 0.01, ***p < 0.001 and ****p < 0.0001. ImageJ was used to improve image quality for better visualisation.

The adsorption assay showed that ∼91% of phage particles adsorbed to the WT strain within 15 min. In contrast, all phage‐resistant mutants failed to support phage adsorption (Figure 3E). Additionally, fitness costs accompanying phage resistance were investigated in relation to bacterial motility, biofilm formation and pyocyanin production. Compared to the WT strain, the phage‐resistant mutants exhibited a loss of twitching and swarming motilities, except for the R14 strain, which showed reduced swarming motility (Figure 4A,B). TEM analysis revealed that the WT strain possessed both type IV pili (T4P) and flagella on its outer surface. In contrast, all phage‐resistant mutants were devoid of these appendages, except for the R14 strain, which was also T4P‐deficient but retained flagella (Figure 4C). In addition, the biofilm‐forming capacity of the phage‐resistant mutants was remarkably reduced by approximately 1.8‐fold (Figure 4D). Moreover, the phage‐resistant mutants exhibited lowered pyocyanin production. The percentage reductions were about 85.9%, 44.6% and 79.1% in the R3, R6 and R14 strains, respectively (Figure 4E). These findings indicate that phage resistance incurred a substantial trade‐off in virulence.

### 3.3 Changes in Antimicrobial Susceptibility and In Vivo Assays of Phage‐Resistant Mutants

**FIGURE 5:**
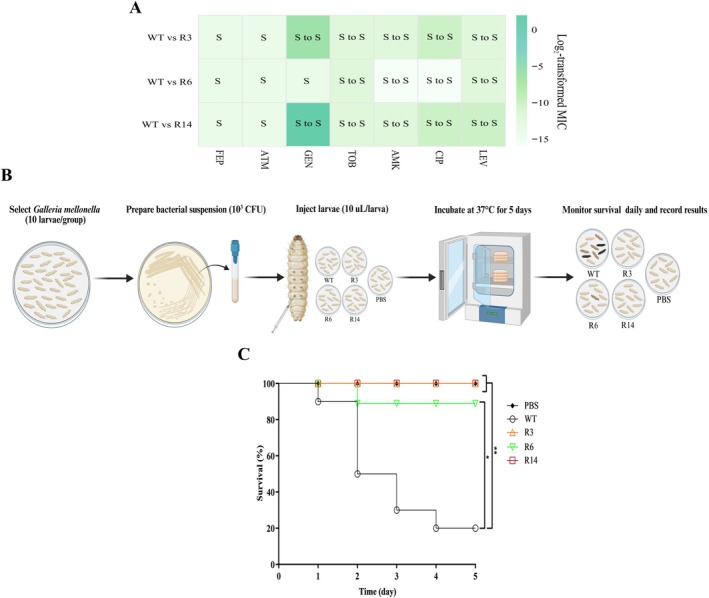
Antibiotic susceptibility and survival assays. (A) The MICs of seven antibiotics were measured using the microbroth‐dilution method between the wild type (WT) strain and the phage‐resistant mutants (R3, R6 and R14). AMK, amikacin; ATM, aztreonam; CIP, ciprofloxacin; FEP, cefepime; GEN, gentamicin; LEV, levofloxacin; R, resistant; S, sensitive; TOB, tobramycin. (B) Schematic of *G. mellonella* infection model. (C) The Kaplan–Meier survival curve of *G. mellonella* challenged with the WT strain or its mutants. The log‐rank (Mantel–Cox) test was used for statistical analysis. *p < 0.05, **p < 0.01.

Considering the potential interplay between phage resistance and antibiotic sensitivity, we performed antibiotic susceptibility testing on the WT strain and its phage‐resistant mutants using the microbroth‐dilution method. Compared with the WT strain, the R3 and R14 strains had 8× and 16× decreases (GEN), 2× decreases (AMK), 4× decreases (CIP), and 2× and 4× decreases (LEV), respectively, in their MICs. The R6 strain showed no change in MIC for GEN, whereas it demonstrated a 2× increase (AMK and CIP) and a 2× decrease (LEV). Notably, all resistant mutants became two times more susceptible to TOB. None of the tested strains showed differences in MICs of the remaining antibiotics (Figure 5A).

As a fitness cost, phage resistance evolution may result in changes in bacterial virulence inside the host. To compare the virulence between the WT strain and its phage‐resistant mutants, we employed *G. mellonella*, which presents a cost‐effective and ethically favourable model for virulence assays. Notably, the survival rates of the larvae injected with the phage‐resistant mutants were higher than that of the WT strain. All larvae injected with the R3 and R14 strains maintained 100% survival 5 days post‐inoculation, whereas the survival rates of R6‐injected larvae remained at 90%. In contrast, only 20% of the larvae survived when injected with the WT strain. These results suggest that phage resistance entailed diminished virulence in vivo as a fitness cost (Figure 5B,C).

**FIGURE 6:**
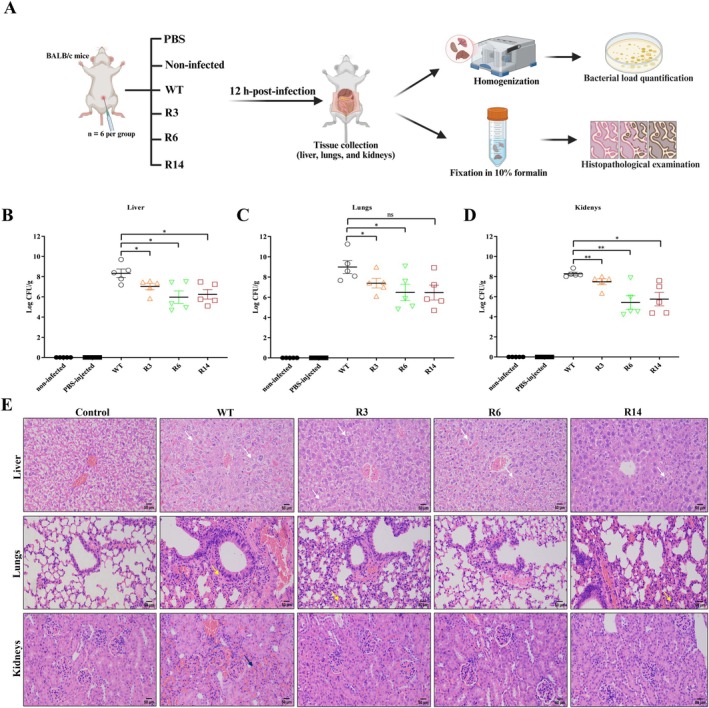
Colonisation and pathogenicity assays of the wild‐type (WT) strain and the phage‐resistant mutants (R3, R6 and R14). (A) Schematic of mouse infection model. Bacterial loads within (B) liver, (C) lungs and (D) kidneys of mice at 12 h after infection with the WT strain or its mutants. The mice tissues were aseptically retrieved and homogenised for bacterial enumeration. (E) Histopathological examination of haematoxylin and eosin (H and E)‐stained mice tissues post‐infection with the WT strain or its mutants. Control groups displayed normal tissue architecture. The WT‐infected liver sections displayed many hepatocytes with evident structural blurring with focal karyorrhexis, while only a few hepatocytes in the phage‐resistant mutants had shrunk and disappeared, with slight blurring (white arrows). The lung tissues of the WT‐infected group showed marked alveolar septal thickening, in contrast to mild thickening in those of the phage‐resistant mutants (yellow arrows). The WT‐infected kidney sections exhibited prominent haemorrhage present in the interstitium (black arrow); however, the phage‐resistant mutant showed normal kidney histology. *p < 0.05, **p < 0.01. Scale bar, 50 μm.

Next, we assessed the ability of phage‐resistant mutants to colonise and their pathogenicity in mouse tissues to better mimic mammalian infection and immune responses. Compared to the WT‐infected group, mice infected with the phage‐resistant mutants displayed lower bacterial burdens within their tissue organs. The R3‐injected mouse group exhibited a reduction in bacterial loads of 1.31, 1.59 and 0.8 log CFU in the liver, lungs and kidneys, respectively. Of note, a more than 2‐log CFU reduction was observed in tissue organs isolated from the R6‐ or R14‐injected mice groups (Figure 6A–D). Therefore, the phage‐resistant mutants maintained a lower ability to colonise mice compared with the WT strain, suggesting reduced in vivo fitness.

To gain insight into host tissue responses after infection with the WT strain or its phage‐resistant mutants, histopathological examination was conducted. Control groups displayed normal tissue architecture with intact hepatocytes, alveolar spaces and renal tubules. In contrast, the liver tissues of mice infected with the WT strain exhibited significant abnormalities, characterised by more extensive cytoplasmic clearing within the hepatocytes compared to the phage‐resistant mutant strains. Additionally, numerous hepatocytes exhibiting structural blurring and focal karyorrhexis were observed in the WT‐infected group. Meanwhile, only a small number of hepatocytes in the phage‐resistant mutants showed atrophy and disappearance, along with slight blurring of their boundaries (Figure 6E).

Moreover, the WT‐infected lung tissues showed marked pathologic changes, including progressive alveolar obliteration and thickening of the interalveolar septum, with proliferative connective tissue observed around some airways. Conversely, the phage‐resistant mutants exhibited mild lung damage, including uniform alveolar size, relatively normal tracheal epithelium, slight alveolar septal thickening and minimal inflammatory infiltration. Furthermore, the interstitial haemorrhage was more pronounced in renal tissues of the WT‐infected group. On the other hand, the phage‐resistant‐infected groups did not exhibit significant abnormalities in the glomeruli, and the overall histology appears within normal limits (Figure 6E). These findings indicate that phage resistance evolution conferred a fitness defect manifested as reduced pathogenicity in mice.

### 3.4 Comparative Genomic Analysis

To further reveal mutations underlying phage resistance and the fitness cost of phage‐resistant mutants (R3, R6 and R14), whole‐genome resequencing was performed. The results revealed multiple insertion–deletion (indel) mutations and single nucleotide polymorphisms (SNPs) (Tables S4–S6). Specifically, the R3 and R14 strains harboured missense mutations in PA5040 (*pilQ*, encoding a fimbrial assembly protein), while the R6 strain carried a missense mutation in PA4547 (*pilR*, which functions as a cytoplasmic response regulator); both genes are crucial for T4P biosynthesis and function, and mutations in either are predicted to impose phage resistance. Additionally, the R6 strain exhibited a frameshift mutation in PA2023 (*galU*, involved in LPS production) and a missense mutation in PA3802 (*hisS*, associated with histidyl‐tRNA ligase biosynthesis). The R14 strain showed a synonymous mutation in PA0192 (*tonB*, which encodes an energy‐transducing protein) besides a missense mutation in PA4402 (*argJ*, coding for the arginine biosynthesis bifunctional protein).

### 3.5 Transcriptome and RT‐qPCR Analyses

**FIGURE 7:**
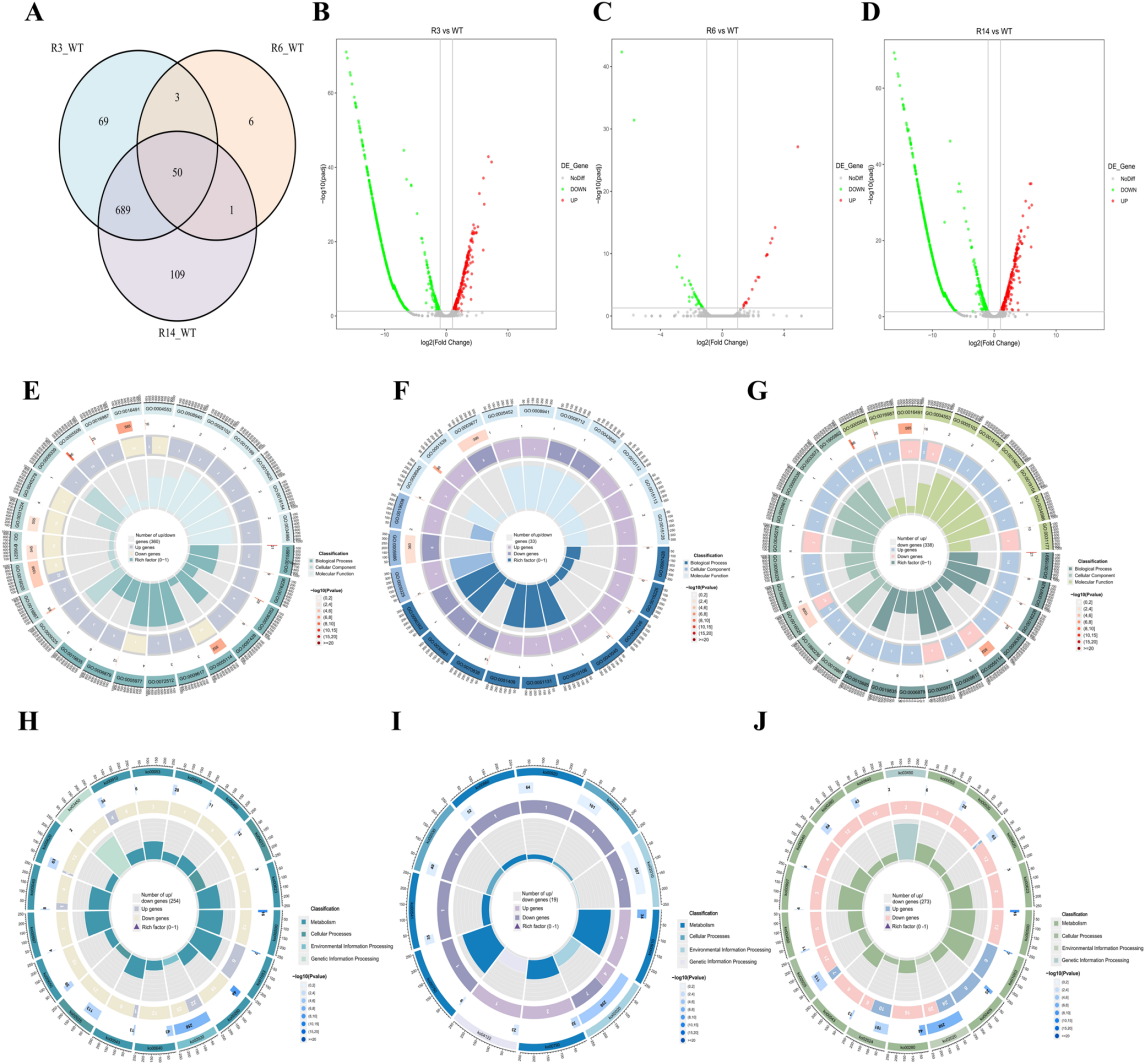
Transcriptome analysis of the wild type (WT) strain and the phage‐resistant mutants (R3, R6 and R14). (A) Venn diagrams illustrating the shared and unique distribution of expressed genes among the WT strain and the R3, R6 and R14. (B–D) Volcano plots showing upregulated (red dots) and downregulated (green dots) differentially expressed genes (DEGs). The default criteria for identifying significantly DEGs were an FDR < 0.05 and an absolute log2 fold change (|log2 FC|) ≥ 1. (E–G) GO enrichment. (H–J) KEGG enrichment overview.

Transcriptomic analysis using RNA sequencing was conducted to explore global gene expression changes and regulatory pathways associated with resistance acquisition and the obtained fitness costs. The results revealed that, compared to WT, the R3, R6 and R14 strains shared 69, 6 and 109 differentially expressed genes (DEGs), respectively (Figure 7A).

Compared with WT, the resistant strain R3 showed 242 upregulated and 569 downregulated DEGs (Figure 7B). Gene Ontology (GO) enrichment analysis included 360 genes, of which 98 were upregulated (27.22%) and 262 were downregulated (72.78%) (Figure 7E). GO analysis of the upregulated DEGs indicated enrichment in iron acquisition and metabolism, outer membrane and transmembrane transport, transcriptional regulation, nitrogen metabolism and bacterial stress and defence responses (Figure S1A). Downregulated genes were significantly enriched in oxidation–reduction processes, glycogen metabolism, membrane structure and transport, osmotic regulation and amino acid catabolism (Figure S1B). KEGG enrichment analysis involved 254 genes, with 62 upregulated (24.4%) and 192 downregulated (75.6%) (Figure 7H). Upregulated DEGs were mainly associated with iron uptake (siderophore synthesis), environmental signal perception and transduction (two‐component systems), and alternative respiration (nitrogen metabolism) (Figure S1C). Downregulated genes were primarily enriched in carbohydrate metabolism, amino acid degradation, biofilm formation, xenobiotic degradation and nitrogen metabolism (Figure S1D).

The resistant strain R6 exhibited 19 upregulated and 41 downregulated DEGs compared to WT (Figure 7C). GO enrichment analysis included 33 genes, with 12 upregulated (36.36%) and 21 downregulated (63.64%) (Figure 7F). Upregulated DEGs were mainly enriched in nitrate reduction (anaerobic respiration), iron–sulfur cluster assembly and iron starvation response, and molybdenum cofactor metabolism (Figure S2A). Downregulated DEGs were primarily associated with transcriptional regulation, ion transport, stress response and redox metabolism (Figure S2B). KEGG enrichment analysis involved 19 genes, with 7 upregulated (36.84%) and 12 downregulated (63.16%) (Figure 7I). Upregulated DEGs were mainly enriched in iron uptake (siderophore synthesis), environmental signal perception and transduction (two‐component systems) and alternative respiration (nitrogen metabolism) (Figure S2C). Downregulated genes were significantly enriched in the two‐component signal transduction system (Figure S2D).

Compared to WT, the resistant strain R14 showed 272 upregulated and 577 downregulated DEGs (Figure 7D). GO enrichment analysis included 338 genes, with 112 upregulated (33.14%) and 226 downregulated (66.86%) (Figure 7G). Upregulated DEGs were mainly enriched in nitrate reduction (anaerobic respiration), iron–sulfur cluster assembly and iron starvation response, and molybdenum cofactor metabolism (Figure S3A). Downregulated DEGs were primarily associated with transcriptional regulation, ion transport, stress response and redox metabolism (Figure S3B). KEGG enrichment analysis involved 273 genes, with 70 upregulated (25.64%) and 203 downregulated (74.36%) (Figure 7J). Upregulated DEGs were mainly enriched in siderophore and phenazine synthesis, two‐component signal systems and quorum sensing (Figure S3C). Downregulated genes were enriched in metabolic pathways, particularly carbohydrate and amino acid metabolism (Figure S3D).

**FIGURE 8:**
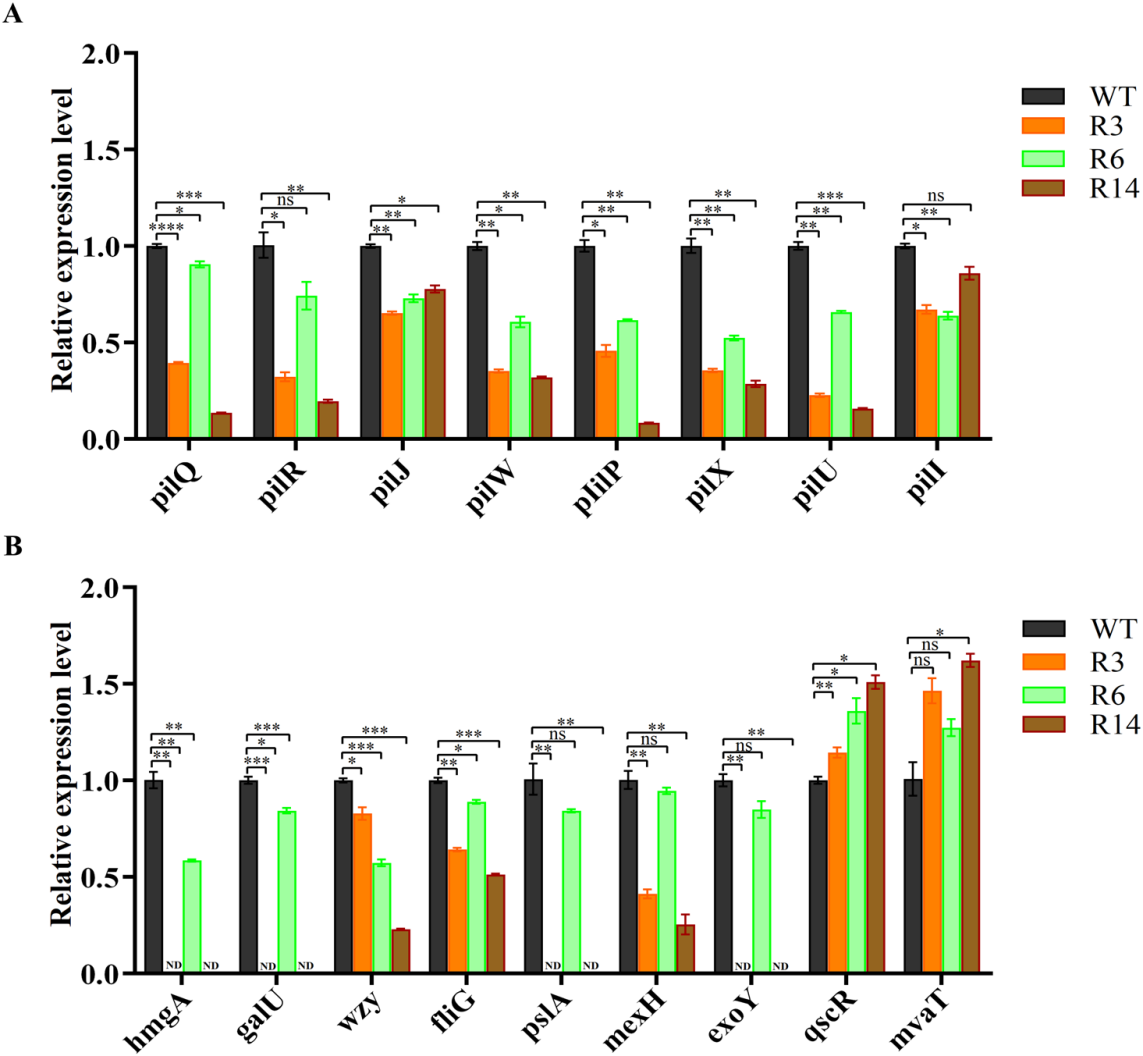
RT‐qPCR validation of differentially expressed key genes of the wild type (WT) strain and the phage‐resistant mutants (R3, R6 and R14). (A) Expression levels of type IV pili‐related genes. (B) Expression levels of other selected genes. *p < 0.05, **p < 0.01, ***p < 0.001, ****p < 0.0001. ND, not detected.

RT‐qPCR validation of some selected key DEGs, including T4P‐related genes (*pilQ*, *pilR*, *pilJ*, *pilW*, *pilP*, *pilX*, *pilU* and *pilI*), in addition to other genes associated with fitness trade‐offs (*hmgA*, *galU*, *wzy*, *fliG*, *pslA*, *mexH*, *exoY*, *qscR* and *mvaT*), demonstrated that the trends aligned with the RNA sequencing analysis (Figure 8A,B).

## 4 Discussion

The emergence of multidrug‐resistant *Pseudomonas aeruginosa* strains, together with the lack of new antibiotic discoveries, has renewed the interest in phage therapy. In this study, a new lytic Henu5 phage that targets *Pseudomonas aeruginosa* was isolated from sewage. The isolated phage formed clear plaques, signifying its lytic nature. Plaques were surrounded by hazy halo zones, suggesting exopolysaccharide depolymerase activity (Panteleev et al. 2025). TEM examination parallel with genomic analysis indicated that phage Henu5 belongs to the *Pakpunavirus* genus within the *Caudoviricetes* class. For industrial applications, environmental changes, such as pH and temperature, have been reported to influence phage stability and infectivity (Wdowiak et al. 2022). Moreover, chloroform is often added during purification, enrichment and production of phage particles (Hietala et al. 2019; Hyman 2019). Notably, the observed phage Henu5 stability across varying environmental conditions highlights its robustness and supports its potential for use in industrial settings. A detailed phage genome analysis allowed the detection of 17 tRNAs, which most likely correspond to codons that are crucial for phage translation and propagation (Bailly‐Bechet et al. 2007). One of the key limitations of phage therapy is the possibility that phages may contribute to the emergence of antibiotic resistance and their capacity for lysogeny (Principi et al. 2019). Importantly, the phage Henu5 genome was devoid of genes encoding antibiotic resistance, virulence factors or integrases, hence eliminating these concerns.

The rapid development of phage‐resistant bacteria poses a serious threat to the successful application of phages (Schooley et al. 2017). Phage selective pressure induces mutations in the bacterial genome, allowing them to counteract phage infection. Nevertheless, this often imposes a fitness cost, uncovering new perspectives for advancing phage therapy (Xuan et al. 2022). In this regard, exploring phage resistance and the accompanying fitness trade‐offs in bacterial pathogens will help improve the phage treatment outcomes. In the present study, during phage Henu5 and *Pseudomonas aeruginosa* co‐evolution, *Pseudomonas aeruginosa* developed phage resistance. Interestingly, the phage‐resistant mutants displayed two types: brown and white colony phenotypes, with a white phenotype occurring at a higher frequency, as reported by (Shen et al. 2018). For further experiments, we selected two strains producing a brown pigment (R3 and R14) and one strain with a white colony phenotype (R6). Of note, the phage‐resistant mutants displayed altered colony morphology, accompanied by changes in growth rates, reflecting an adaptive trade‐off, as previously reported (Wannasrichan et al. 2022).

Bacteria have been reported to evolve phage resistance via adsorption inhibition mechanisms, including modifications or loss of phage‐specific receptors. Type IV pili (T4P) are not only considered virulence factors for *Pseudomonas aeruginosa* but also serve as primary phage receptors (Harvey et al. 2018). The T4P are made of a multiprotein complex spanning across the cell envelope, including PilR and PilQ proteins. PilR is reported to control *pilA* expression, the major subunit of T4P, while PilQ, encoded by *pilQ*, forms an outer membrane pore through which T4P exits the cell (Burrows 2012). *PilQ* mutants have been previously reported to lack twitching motility and exhibit resistance to pilus‐specific phages (Martin et al. 1993). In this study, comparative genome analysis identified missense mutations in the *pilQ* gene (R3 and R14) and the *pilR* gene (R6). As expected, all resistant mutants showed a lack of twitching motility on agar plates and a phage‐resistant phenotype. Together, we inferred that the detected mutations are presumably required for T4P function and phage infection.

Additionally, T4P has been reported to regulate swarming motility and biofilm formation (Li et al. 2022). Accordingly, we sought to evaluate these phenotypic traits in the WT strain and phage‐resistant mutants. In contrast to the abolished swarming motility observed with the R3 and R6 strains compared with the WT strain, the R14 strain exhibited a decrease in the distance migrated, which was further explained by the whole‐genome resequencing, revealing an additional missense mutation in a*rgJ*, a gene involved in arginine biosynthesis (Yee et al. 2017). Arginine has been previously reported to completely repress swarming motility and promote biofilm formation in *Pseudomonas aeruginosa* (Kohler et al. 2000; Bernier et al. 2011). These findings indicate that disruption of *argJ* partially compensates for the absence of pilus‐mediated movement, allowing limited swarming. Moreover, TEM confirmed the altered motility patterns, supporting the involvement of surface structural changes in the observed phenotypes. In keeping with twitching motility findings, the observed reduced biofilm phenotype in our phage‐resistant mutants implies the key role of T4P in biofilm development (Ochner et al. 2024). Collectively, our data indicate that phage selection induces evolutionary fitness costs as exhibited by reduced virulence.

The ability of *Pseudomonas aeruginosa* to produce biofilms poses a serious threat to public health owing to its robust ability to colonise biomaterials and confer resistance to the antimicrobials (Høiby et al. 2011). In line with this, our phage‐resistant mutants with impaired biofilm formation exhibited hypersensitivity to certain antibiotics compared to the WT strain (Figure 5A), aligned with (Markwitz et al. 2021). Another plausible explanation is that T4P might help the bacterial host alter collective motion, enabling escape from antimicrobial compounds. Consistent with this, the *pilA* mutant has been reported to exhibit increased susceptibility to pulmonary innate immunity surfactant protein‐A (SP‐A)‐mediated bacterial opsonisation and membrane permeabilisation (Tan et al. 2014; Li et al. 2022). It is noteworthy that Chan et al. exploited phage resistance to re‐sensitise pathogenic bacteria to antibiotics when the phage receptor was also an antibiotic efflux pump, thereby proposing phage‐antibiotic combination therapy in light of these findings (Chan et al. 2018). Moreover, a clinical trial reported by (Bao et al. 2020) revealed that a combination of sulfamethoxazole–trimethoprim with a phage cocktail has successfully cured a patient with a recurrent urinary tract infection caused by extensive drug‐resistant *Klebsiella pneumoniae* despite in vitro resistance to this antibiotic. Therefore, we postulated that the acquisition of phage resistance imposes a fitness trade‐off in antibiotic susceptibility, which may inspire the use of phage‐antibiotic combination treatment as a potential therapeutic approach.

Phage‐antibiotic synergy (PAS) has been increasingly recognised as a potentially valuable approach with multiple therapeutic benefits. A Belgian consortium has recently reported *Pseudomonas aeruginosa* as the most common pathogen treated with phage therapy (accounting for 49/100 cases). Nevertheless, across all targeted pathogens, about 69.3% of patients received phages and antibiotics simultaneously, and the data showed that bacterial clearance was remarkably reduced when antibiotics were used alone without phages (Pirnay et al. 2024). Moreover, PAS can reduce the antibiotic dosage, thereby mitigating the side effects of some toxic drugs, such as polymyxin B, for last resort cases (Fatima and Hynes 2025). Furthermore, PAS can effectively eliminate dormant ‘persister’ cells in biofilm that survive standard antibiotic treatments, offering a promising approach against recurrent *Pseudomonas aeruginosa* infections (Maffei et al. 2024). It is noteworthy that phages have been recently reported as a fundamental tool to explore novel anti‐virulence agents. Shimozono et al. reported the use of *Pseudomonas aeruginosa* in combination with a T4P‐targeting phage, φKMV, to screen for novel T4P inhibitors. This led to the identification of tuspetinib, depending on its ability to inhibit bacterial lysis by its phage. This approach allows for rapid identification of new anti‐virulence compounds that can be used in combination with traditional antibiotics against resistant infections (Shimozono et al. 2025).

Besides, previous studies have reported the implication of T4P in *Pseudomonas aeruginosa* pathogenicity and the establishment of infections (Persat et al. 2015). Similarly, using *G. mellonella* and mouse infection models, our phage‐resistant mutants demonstrated remarkable survival rates, reduced bacterial burdens and normal histology compared to the WT strain. Hence, we speculate that the phage‐resistant mutants displayed substantial defects in infectivity while being more prone to immune clearance and, consequently, less fit for colonisation and systemic dissemination than the WT strain (Chen et al. 2024).

Genomic analysis revealed additional mutations, including those in the *galU* gene in the R6 strain and both the *tonB* and *argJ* genes in the R14 strain. The *galU* gene has been previously reported to be associated with the brown colony phenotype (Shen et al. 2018), and it has also been linked to bacterial growth, adherence and in vivo pathogenicity in *Streptococcus pneumoniae* (Cools et al. 2018). Additionally, the *galU* mutant *Salmonella Typhimurium* has been shown to be defective in biofilm formation and motility, along with enhanced antibiotic sensitivity and attenuated pathogenicity in animal models (Guo et al. 2023). Moreover, the TonB protein plays crucial roles in iron acquisition in *Pseudomonas aeruginosa* , and mutation in its encoding gene could impair iron uptake, resulting in impaired in vivo growth and virulence (Takase et al. 2000). A mutation in *argJ* in *Staphylococcus aureus* caused comparable in vivo outcomes (Yee et al. 2017). Taken together, in addition to previously described roles of T4P, additional mutations identified in this study might also contribute to the adaptive trade‐offs.

Enrichment analysis combining GO terms and KEGG pathways revealed that the downregulated genes across all resistant mutants (R3, R6 and R14) were primarily associated with a core set of metabolic and regulatory processes. These included carbohydrate and amino acid metabolism, transcriptional regulation, ion transport, redox homeostasis and stress response pathways. These transcriptional downregulations might undermine bacterial ability to acquire nutrients, adapt to the host environment, coordinate virulence factor production and withstand immune attacks, resulting in attenuated virulence and pathogenicity during infection. Together these alterations are presumably responsible for the observed fitness costs accompanying phage resistance. Additionally, RT‐qPCR analysis of the expression of selected key DEGs further validated RNA sequencing. Despite the current study presenting a comprehensive dataset including phage biology, genomics, bacterial phenotyping, animal models and multi‐omics analyses, there are some limitations. Further investigation involving the inclusion of knockouts, complementation or genetic reconstruction experiments is required to support causal links between specific mutations and observed phenotypes, including phage resistance, biofilm reduction and antibiotic susceptibility changes.

In conclusion, phage Henu5 is a novel lytic phage targeting *Pseudomonas aeruginosa* belonging to the *Pakpunavirus* genus within the *Caudoviricetes* class. Henu5 shows good physicochemical stability, and its genome is devoid of integrases, resistance or virulence genes, further confirming its safety as a potential therapeutic agent. Mutations of the T4P‐related genes are assumed to impair phage adsorption and lead to phage‐resistant phenotypes. Besides, these mutations, together with the other genetic changes and the wide transcriptional alterations, are likely responsible for the trade‐offs obtained in bacterial growth, virulence and antibiotic susceptibility, along with in vivo fitness. Collectively, this study integrated phenotypic, genomic and transcriptomic analyses of phage‐driven resistance and the potential fitness costs that might direct future phage selection in clinical settings.

## Author Contributions

**Jingjing Li:** methodology, software, formal analysis, investigation, data curation, writing – original draft, writing – review and editing. **Chaonan Ma:** writing – review and editing, investigation. **Guanbin Qi:** writing – review and editing, project administration. **Salwa E. Gomaa:** conceptualization, methodology, validation, software, formal analysis, data curation, investigation, visualization, writing – review and editing, writing – original draft. **Tieshan Teng:** writing – review and editing, resources, supervision, project administration, funding acquisition. **Qiming Li:** project administration, investigation. **Yangyang Liu:** writing – review and editing, data curation. **Jiawen Shen:** software, writing – review and editing.

## Funding

This work was supported by Henan Provincial Science and Technology Research Project (LHGJ20250513) and the Key R&D and Promotion Projects of Henan Province (262102311032 and 262102311177).

## Ethics Statement

The animal experiment was reviewed and approved by Henan University's Subcommittee on Biomedical Research Ethics (Approval number: HUSOM2025‐765).

## Conflicts of Interest

The authors declare no conflicts of interest.

## Supporting information


**Figure S1:** Transcriptome analysis of the wild‐type (WT) strain and the phage‐resistant mutant (R3). (A and B) GO enrichment of analysis with upregulated downregulated and differentially expressed genes (DEGs). (C and D) KEGG enrichment analysis with upregulated and downregulated DEGs.
**Figure S2:** Transcriptome analysis of the wild‐type (WT) strain and the phage‐resistant mutant (R6). (A and B) GO enrichment of analysis with upregulated downregulated and differentially expressed genes (DEGs). (C and D) KEGG enrichment analysis with upregulated and downregulated DEGs.
**Figure S3:** Transcriptome analysis of the wild‐type (WT) strain and the phage‐resistant mutant (R14). (A and B) GO enrichment of analysis with upregulated downregulated and differentially expressed genes (DEGs). (C and D) KEGG enrichment analysis with upregulated and downregulated DEGs.

**Table S1:** Primers for qRT‐PCR.
**Table S2:** Detailed genome annotation of Henu5 phage.
**Table S3:** Details of tRNAs predicted in the Henu5 genome.
**Table S4:** Identified mutations in the genome of the R3 mutant using *P. aeruginosa* PAO1 as reference.
**Table S5:** Identified mutations in the genome of the R6 mutant using *P. aeruginosa* PAO1 as reference.
**Table S6:** Identified mutations in the genome of the R14 mutant using *P. aeruginosa* PAO1 as reference.

## Data Availability

The complete genome sequence of *Pseudomonas aeruginosa* phage Henu5 has been submitted to GenBank under accession number NC_073608.1. All other data generated or analysed during this study are included in this article and its Supporting Information or are available from the corresponding author upon reasonable request.
