# Supplementary material for: Isolation of a Novel Lytic Pseudomonas aeruginosa Phage Henu5 and Fitness Costs of Phage‐Driven Resistance
Source: Microb Biotechnol. 2026 May 28;19(6):e70389. doi: 10.1111/1751-7915.70389 (PMC13238721; doi:10.1111/1751-7915.70389)
Supplement: Supplementary file 1 — Figure S1: Transcriptome analysis of the wild‐type (WT) strain and the phage‐resistant mutant (R3). (A and B) GO enrichment of analysis with upregulated downregulated and differentially expressed genes (DEGs). (C and D) KEGG enrichment analysis with upregulated and downregulated DEGs. Figure S2: Transcriptome analysis of the wild‐type (WT) strain and the phage‐resistant mutant (R6). (A and B) GO enrichment of analysis with upregulated downregulated and differentially expressed genes (DEGs). (C and D) KEGG enrichment analysis with upregulated and downregulated DEGs. Figure S3: Transcriptome analysis of the wild‐type (WT) strain and the phage‐resistant mutant (R14). (A and B) GO enrichment of analysis with upregulated downregulated and differentially expressed genes (DEGs). (C and D) KEGG enrichment analysis with upregulated and downregulated DEGs. [file MBT2-19-e70389-s002.pdf]

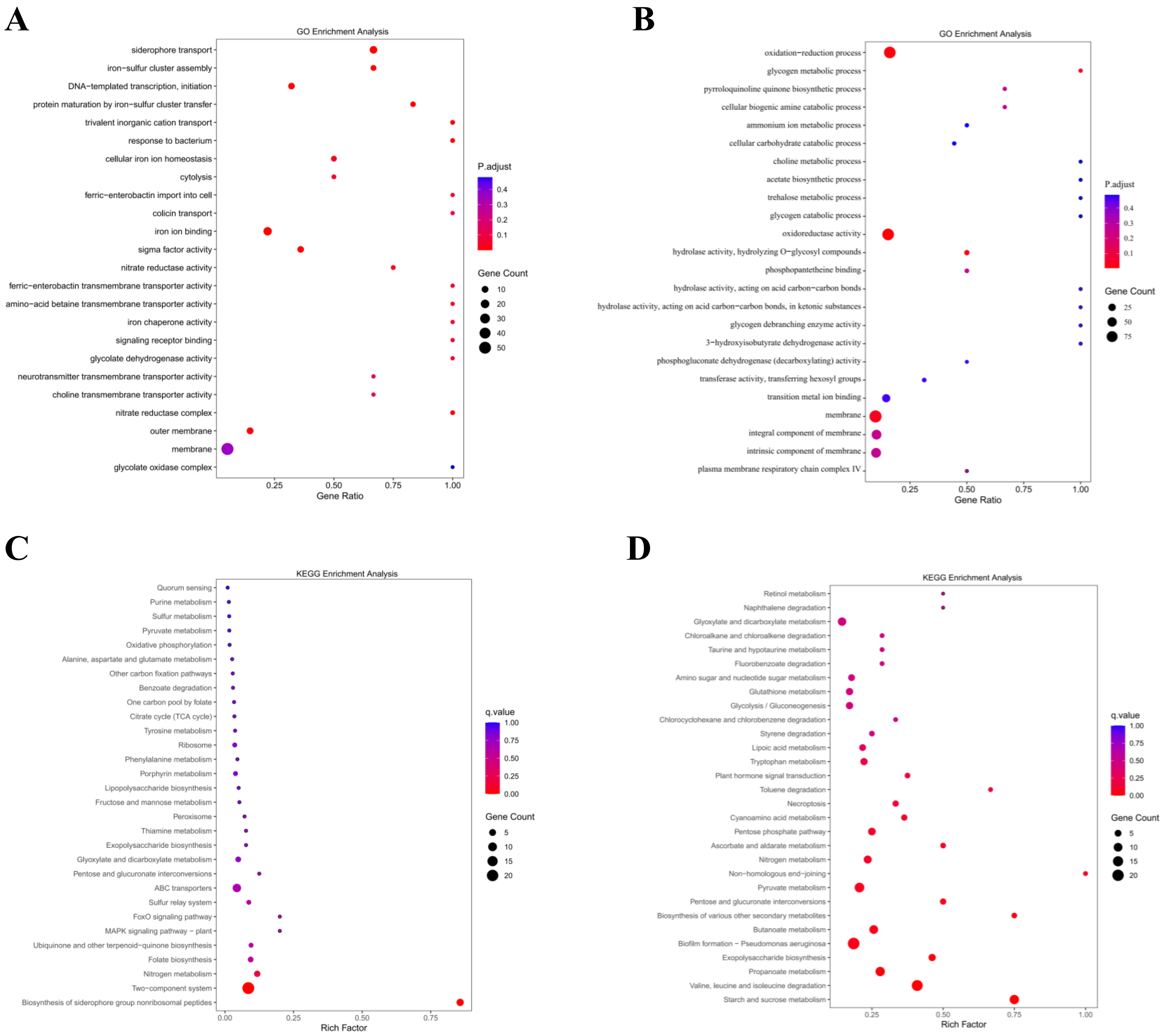

Fig. S1. Transcriptome analysis of the wild-type (WT) strain and the phage-resistant mutant (R3). (A and B) GO enrichment of analysis with upregulated and downregulated differentially expressed genes (DEGs). (C and D) KEGG enrichment analysis with upregulated and downregulated DEGs.

Fig. S2. Transcriptome analysis of the wild-type (WT) strain and the phage-resistant mutant (R6). (A and B) GO enrichment of analysis with upregulated and downregulated differentially expressed genes (DEGs). (C and D) KEGG enrichment analysis with upregulated and downregulated DEGs.

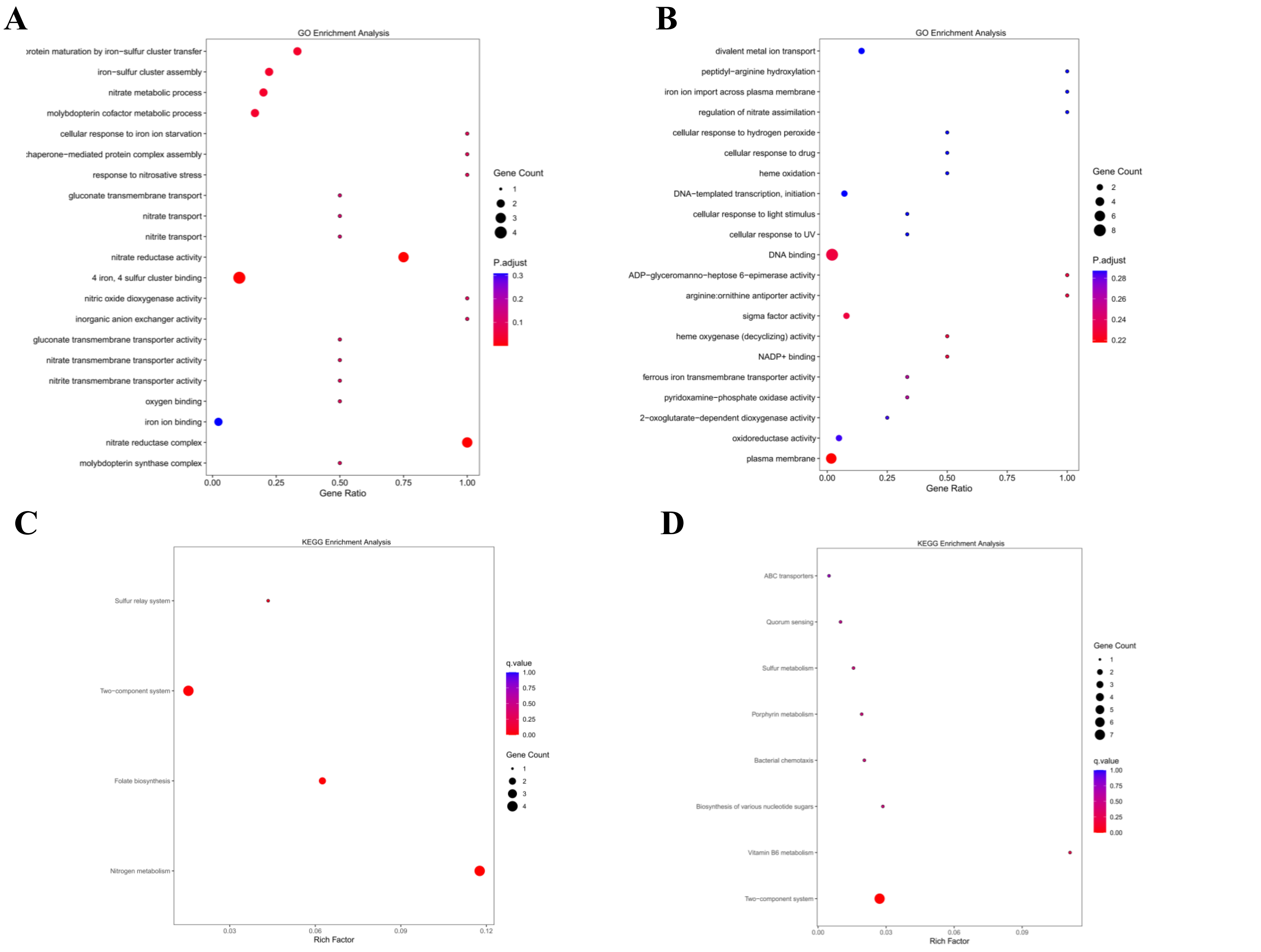

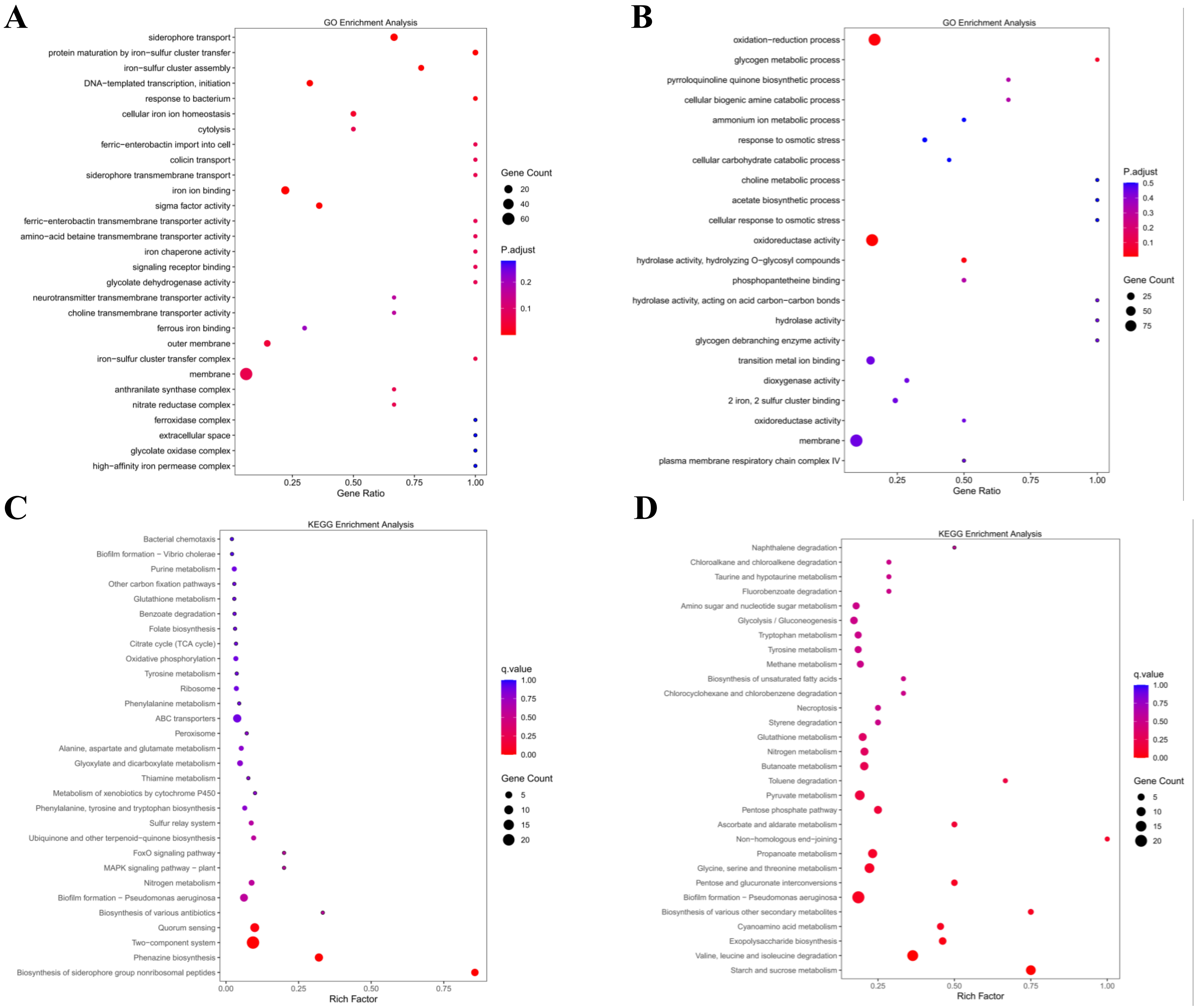

Fig. S3. Transcriptome analysis of the wild-type (WT) strain and the phage-resistant mutant (R14). (A and B) GO enrichment of analysis with upregulated and downregulated differentially expressed genes (DEGs). (C and D) KEGG enrichment analysis with upregulated and downregulated DEGs.
