# Supplementary material for: Isolation of a Novel Lytic Pseudomonas aeruginosa Phage Henu5 and Fitness Costs of Phage‐Driven Resistance
Source: Microb Biotechnol. 2026 May 28;19(6):e70389. doi: 10.1111/1751-7915.70389 (PMC13238721; doi:10.1111/1751-7915.70389)
Supplement: Supplementary file 2 — Table S1: Primers for qRT‐PCR. Table S2: Detailed genome annotation of Henu5 phage. Table S3: Details of tRNAs predicted in the Henu5 genome. Table S4: Identified mutations in the genome of the R3 mutant using P. aeruginosa PAO1 as reference. Table S5: Identified mutations in the genome of the R6 mutant using P. aeruginosa PAO1 as reference. Table S6: Identified mutations in the genome of the R14 mutant using P. aeruginosa PAO1 as reference. [file MBT2-19-e70389-s001.docx]

**Supplementary Table S1:** Primers for qRT-PCR

| **Genes** | **Primers** | **Sequence (5'-3')** |
| --- | --- | --- |
| *pilQ* | pilQ-F | CACGACCTTGGGTTGGGAGA |
|  | pilQ-R | GGATCGGCATCGGCTTCAT |
| *pilR* | pilR-F | TGCCTTCGACTTCCTCACCAA |
|  | pilR-R | CTACCGGACTCGCCACTGAT |
| *pilJ* | pilJ-F | CCAACTTTGCCTACCTCAACACC |
|  | pilJ-R | ACGTCCATCTGCGGCTTCA |
| *pilW* | pilW-F | TTCGTCGAACAGGCCAACAT |
|  | pilW-R | TAGAGGCGGTCCTTGTCGTT |
| *pilP* | pilP-F | TCGGGCTTGATCACCTTGTT |
|  | pilP-R | CCGATCTGCAGTCCTACATGG |
| *pilX* | pilX-F | CGAACAGACTCGCTTGCAGAAC |
|  | pilX-R | CGCGGTAACTCATCCAGGTATT |
| *pilU* | pilU-F | CTTCCGGGTCAGTGCCTTCT |
|  | pilU-R | TGGAGTTCTTGTTGCGGTAGC |
| *pilI* | pilI-F | GCTGCCGATCATGGACCTCT |
|  | pilI-R | AAGACGCCATGAATGAAGGGTT |
| *hmgA* | hmgA-F | CGGGTTGAAGGTGGATACGA |
|  | hmgA-R | ACCGCAACCTGATGAACGAA |
| *galU* | galU-F | GAAATGCTGCCGGTGGTGA |
|  | galU-R | TCGGTGTTGCGGATCTGGT |
| *wzy* | wzy-F | TTTCGACGATTCGGTCAGGT |
|  | wzy-R | AGCTCAACGGCTCAGTCCTACC |
| *fliG* | fliG-F | GCACATGGGACCGAAAGAGG |
|  | fliG-R | GATGCGGTCGATCAGGTTGTT |
| *PslA* | PslA-F | CGATCAGGCTCGACTCCAAG |
|  | PslA-R | ATGCTGGTCTTGCGGATGAA |
| *mexH* | mexH-F | CGGCTCGGCGAAGAAAGA |
|  | mexH-R | GTTCCACCGCGTCGTTGAGT |
| *exoY* | exoY-F | TTGCGTATTTCCGCCGTGT |
|  | exoY-R | TTCCCGACCAAGGGCTTCT |
| *qscR* | qscR-F | ATGCTGCAGGCTACCTTCGG |
|  | qscR-R | CACTTGAGCATCTCGGTTTCC |
| *mvaT* | mvaT-F | TCCCTGCGTGACGTGATTTC |
|  | mvaT-R | CCACTTGGCTTTCCACTCTTTC |
| *rho* | rho-F | GGATGACCACGTCCTTCTTGTG |
|  | rho-R | CTGAGGAAGTGACCGAAATGC |

**Supplementary Table S2:** Detailed genome annotation of Henu5 phage

| **ORF** | **Start** | **Stop** | **Amino acids** | **Strand** | **Molecular weight in KDa** | **Product** | **DbXrefs** |
| --- | --- | --- | --- | --- | --- | --- | --- |
| ORF1 | 76 | 1704 | 542 | + | 61.2 | putative DNA polymerase | GeneID:80099677 |
| ORF2 | 1758 | 2192 | 144 | + | 15.7 | NAD(P)H-dependent oxidoreductase | GeneID:[80099533](https://www.ncbi.nlm.nih.gov/gene/80099533) |
| ORF3 | 2390 | 3100 | 236 | + | 26.3 | hypothetical protein | GeneID:[80099534](https://www.ncbi.nlm.nih.gov/gene/80099534) |
| ORF4 | 3202 | 4206 | 334 | + | 37.1 | hypothetical protein | GeneID:80099535 |
| ORF5 | 4284 | 4517 | 77 | + | 8.4 | hypothetical protein | GeneID:[80099536](https://www.ncbi.nlm.nih.gov/gene/80099536) |
| ORF6 | 4578 | 4994 | 138 | - | 15.6 | hypothetical protein | GeneID:[80099537](https://www.ncbi.nlm.nih.gov/gene/80099537) |
| ORF7 | 4791 | 5843 | 350 | + | 39.9 | hypothetical protein | GeneID:80099538 |
| ORF8 | 5687 | 6205 | 172 | - | 18.9 | hypothetical protein | GeneID:80099540 |
| ORF9 | 5840 | 6403 | 187 | + | 21.6 | hypothetical protein | GeneID:80099539 |
| ORF10 | 6400 | 6798 | 132 | + | 15.2 | hypothetical protein | GeneID:80099541 |
| ORF11 | 6819 | 7025 | 68 | + | 7.6 | hypothetical protein | GeneID:[80099542](https://www.ncbi.nlm.nih.gov/gene/80099542) |
| ORF12 | 7022 | 7459 | 145 | + | 16.4 | hypothetical protein | GeneID:80099543 |
| ORF13 | 7568 | 7807 | 79 | + | 9.3 | hypothetical protein | GeneID:[80099544](https://www.ncbi.nlm.nih.gov/gene/80099544) |
| ORF14 | 7804 | 8583 | 259 | + | 29.3 | hypothetical protein | GeneID:80099545 |
| ORF15 | 8782 | 8991 | 69 | + | 7.5 | hypothetical protein | GeneID:[80099546](https://www.ncbi.nlm.nih.gov/gene/80099546) |
| ORF16 | 9011 | 9346 | 111 | + | 12.5 | hypothetical protein | GeneID:80099547 |
| ORF17 | 9795 | 10508 | 237 | + | 26.4 | 3'-phosphatase, 5'-polynucleotide kinase | GeneID:80099548 |
| ORF18 | 10715 | 11680 | 321 | + | 36.8 | thymidylate synthase | GeneID:80099549 |
| ORF19 | 11683 | 12027 | 114 | + | 13.2 | hypothetical protein | GeneID:80099550 |
| ORF20 | 11954 | 13090 | 378 | + | 43.8 | ribonucleotide-diphosphate reductase beta subunit | GeneID:80099551 |
| ORF21 | 13083 | 14828 | 581 | + | 66.7 | putative ribonucleotide-diphosphate reductase  alpha subunit | GeneID:80099552 |
| ORF22 | 14977 | 15273 | 98 | + | 11.6 | hypothetical protein | GeneID:[80099553](https://www.ncbi.nlm.nih.gov/gene/80099553) |
| ORF23 | 15242 | 15643 | 133 | - | 14.5 | hypothetical protein | GeneID:80099554 |
| ORF24 | 15742 | 16056 | 104 | + | 11.9 | hypothetical protein | GeneID:80099555 |
| ORF25 | 16245 | 16520 | 91 | + | 10.2 | hypothetical protein | GeneID:[80099556](https://www.ncbi.nlm.nih.gov/gene/80099556) |
| ORF26 | 16513 | 16881 | 122 | - | 13.2 | hypothetical protein | GeneID:80099557 |
| ORF27 | 17281 | 17568 | 95 | - | 10.9 | hypothetical protein | GeneID:[80099558](https://www.ncbi.nlm.nih.gov/gene/80099558) |
| ORF28 | 17826 | 18812 | 328 | + | 37.5 | hypothetical protein | GeneID:80099559 |
| ORF29 | 19573 | 19776 | 67 | + | 7.4 | hypothetical protein | GeneID:[80099560](https://www.ncbi.nlm.nih.gov/gene/80099560) |
| ORF30 | 19837 | 20133 | 98 | + | 11.1 | hypothetical protein | GeneID:[80099561](https://www.ncbi.nlm.nih.gov/gene/80099561) |
| ORF31 | 20144 | 20518 | 124 | - | 14.0 | hypothetical protein | GeneID:80099562 |
| ORF32 | 20757 | 21056 | 99 | + | 11.1 | hypothetical protein | GeneID:[80099563](https://www.ncbi.nlm.nih.gov/gene/80099563) |
| ORF33 | 20945 | 21289 | 114 | + | 12.6 | hypothetical protein | GeneID:80099564 |
| ORF34 | 21437 | 21868 | 143 | - | 16.1 | hypothetical protein | GeneID:80099565 |
| ORF35 | 21867 | 22079 | 70 | + | 7.7 | hypothetical protein | GeneID:[80099566](https://www.ncbi.nlm.nih.gov/gene/80099566) |
| ORF36 | 22509 | 22892 | 127 | + | 15.1 | hypothetical protein | GeneID:80099567 |
| ORF37 | 22968 | 23639 | 223 | + | 24.3 | hypothetical protein | GeneID:80099568 |
| ORF38 | 23644 | 23982 | 112 | + | 12.6 | hypothetical protein | GeneID:80099569 |
| ORF39 | 23910 | 24335 | 141 | + | 15.6 | hypothetical protein | GeneID:80099570 |
| ORF40 | 24809 | 25105 | 98 | + | 11.2 | hypothetical protein | GeneID:[80099571](https://www.ncbi.nlm.nih.gov/gene/80099571) |
| ORF41 | 25102 | 25326 | 74 | + | 8.3 | hypothetical protein | GeneID:[80099572](https://www.ncbi.nlm.nih.gov/gene/80099572) |
| ORF42 | 25359 | 25625 | 88 | + | 9.9 | hypothetical protein | GeneID:[80099573](https://www.ncbi.nlm.nih.gov/gene/80099573) |
| ORF43 | 25622 | 26014 | 130 | + | 15.4 | hypothetical protein | GeneID:80099574 |
| ORF44 | 26190 | 26441 | 83 | + | 9.5 | hypothetical protein | GeneID:[80099575](https://www.ncbi.nlm.nih.gov/gene/80099575) |
| ORF45 | 26519 | 26767 | 82 | + | 9.6 | hypothetical protein | GeneID:[80099576](https://www.ncbi.nlm.nih.gov/gene/80099576) |
| ORF46 | 26802 | 27281 | 159 | + | 17.9 | hypothetical protein | GeneID:80099577 |
| ORF47 | 27365 | 27880 | 171 | + | 19.6 | hypothetical protein | GeneID:80099578 |
| ORF48 | 27425 | 28555 | 376 | - | 41.2 | hypothetical protein | GeneID:80099579 |
| ORF49 | 28554 | 28787 | 77 | + | 8.3 | hypothetical protein | GeneID:[80099580](https://www.ncbi.nlm.nih.gov/gene/80099580) |
| ORF50 | 29206 | 29667 | 153 | + | 17.2 | hypothetical protein | GeneID:80099581 |
| ORF51 | 29684 | 29986 | 100 | + | 11.6 | hypothetical protein | GeneID:80099582 |
| ORF52 | 30080 | 30346 | 88 | + | 9.7 | hypothetical protein | GeneID:[80099583](https://www.ncbi.nlm.nih.gov/gene/80099583) |
| ORF53 | 31072 | 31332 | 86 | + | 9.6 | hypothetical protein | GeneID:[80099584](https://www.ncbi.nlm.nih.gov/gene/80099583) |
| ORF54 | 32113 | 32715 | 200 | + | 22.9 | hypothetical protein | GeneID:80099585 |
| ORF55 | 33434 | 33700 | 88 | - | 10.5 | hypothetical protein | GeneID:[80099586](https://www.ncbi.nlm.nih.gov/gene/80099586) |
| ORF56 | 33700 | 34104 | 134 | - | 15.3 | hypothetical protein | GeneID:80099587 |
| ORF57 | 34094 | 34516 | 140 | - | 16.3 | hypothetical protein | GeneID:80099588 |
| ORF58 | 34533 | 35240 | 235 | - | 27.7 | hypothetical protein | GeneID:80099589 |
| ORF59 | 34953 | 35459 | 168 | + | 18.8 | hypothetical protein | GeneID:80099590 |
| ORF60 | 35243 | 35752 | 169 | - | 19.4 | hypothetical protein | GeneID:80099591 |
| ORF61 | 35752 | 36168 | 138 | - | 15.6 | hypothetical protein | GeneID:80099592 |
| ORF62 | 36165 | 36449 | 94 | - | 11.5 | hypothetical protein | GeneID:[80099593](https://www.ncbi.nlm.nih.gov/gene/80099593) |
| ORF63 | 36504 | 36719 | 71 | - | 8.4 | hypothetical protein | GeneID:[80099594](https://www.ncbi.nlm.nih.gov/gene/80099594) |
| ORF64 | 36716 | 37090 | 124 | - | 14.0 | hypothetical protein | GeneID:80099595 |
| ORF65 | 37059 | 37562 | 167 | - | 19.3 | hypothetical protein | GeneID:80099596 |
| ORF66 | 37549 | 37980 | 143 | - | 17.1 | hypothetical protein | GeneID:80099597 |
| ORF67 | 37946 | 38653 | 235 | - | 26.2 | hypothetical protein | GeneID:80099598 |
| ORF68 | 38527 | 38835 | 102 | - | 11.7 | hypothetical protein | GeneID:80099599 |
| ORF69 | 38837 | 39088 | 83 | - | 9.5 | DUF1776 domain-containing protein | GeneID:[80099600](https://www.ncbi.nlm.nih.gov/gene/80099600) |
| ORF70 | 38998 | 39477 | 159 | - | 17.8 | hypothetical protein | GeneID:80099601 |
| ORF71 | 39474 | 39725 | 83 | - | 9.9 | hypothetical protein | GeneID:[80099602](https://www.ncbi.nlm.nih.gov/gene/80099602) |
| ORF72 | 39682 | 41370 | 562 | - | 63.0 | putative nicotinamide phosphoribosyltransferase | GeneID:80099603 |
| ORF73 | 41369 | 41587 | 72 | + | 7.9 | hypothetical protein | GeneID:[80099604](https://www.ncbi.nlm.nih.gov/gene/80099604) |
| ORF74 | 41642 | 42508 | 288 | - | 31.8 | putative ribose-phosphate pyrophosphokinase | GeneID:80099605 |
| ORF75 | 42518 | 42934 | 138 | - | 15.8 | hypothetical protein | GeneID:80099606 |
| ORF76 | 42945 | 43862 | 305 | - | 34.7 | putative RNA ligase 1 and tail attachment protein | GeneID:80099607 |
| ORF77 | 43874 | 44281 | 135 | - | 15.1 | hypothetical protein | GeneID:80099608 |
| ORF78 | 44320 | 44676 | 118 | + | 13.4 | hypothetical protein | GeneID:80099609 |
| ORF79 | 44552 | 45073 | 173 | + | 20.1 | hypothetical protein | GeneID:80099610 |
| ORF80 | 45704 | 46264 | 186 | - | 21.1 | putative phosphohydrolase | GeneID:80099611 |
| ORF81 | 46266 | 46826 | 186 | - | 21.5 | putative cell wall hydrolase | GeneID:80099612 |
| ORF82 | 46891 | 47352 | 153 | - | 17.2 | hypothetical protein | GeneID:80099613 |
| ORF83 | 47365 | 48573 | 402 | - | 46.1 | putative DNA ligase | GeneID:80099614 |
| ORF84 | 48662 | 48898 | 78 | - | 8.4 | putative CMP/dCMP deaminase | GeneID:80099615 |
| ORF85 | 48928 | 49164 | 78 | - | 8.9 | hypothetical protein | GeneID:80099616 |
| ORF86 | 49174 | 49386 | 70 | - | 7.5 | hypothetical protein | GeneID:[80099617](https://www.ncbi.nlm.nih.gov/gene/80099617) |
| ORF87 | 49383 | 49652 | 89 | - | 10.0 | hypothetical protein | GeneID:[80099618](https://www.ncbi.nlm.nih.gov/gene/80099618) |
| ORF88 | 49661 | 49978 | 105 | - | 12.0 | hypothetical protein | GeneID:80099619 |
| ORF89 | 49992 | 50270 | 92 | - | 10.4 | hypothetical protein | GeneID:[80099620](https://www.ncbi.nlm.nih.gov/gene/80099620) |
| ORF90 | 50267 | 50698 | 143 | - | 15.9 | hypothetical protein | GeneID:[80099621](https://www.ncbi.nlm.nih.gov/gene/80099621) |
| ORF91 | 50634 | 51077 | 147 | + | 16.2 | hypothetical protein | GeneID:[80099622](https://www.ncbi.nlm.nih.gov/gene/80099622) |
| ORF92 | 51235 | 51783 | 182 | - | 20.4 | putative protease subunit | GeneID:[80099624](https://www.ncbi.nlm.nih.gov/gene/80099624) |
| ORF93 | 51831 | 52187 | 118 | - | 13.0 | hypothetical protein | GeneID:[80099623](https://www.ncbi.nlm.nih.gov/gene/80099623) |
| ORF94 | 52184 | 52651 | 155 | - | 18.1 | hypothetical protein | GeneID:[80099625](https://www.ncbi.nlm.nih.gov/gene/80099625) |
| ORF95 | 53457 | 53780 | 107 | + | 12.0 | hypothetical protein | GeneID:[80099626](https://www.ncbi.nlm.nih.gov/gene/80099626) |
| ORF96 | 55979 | 56494 | 171 | + | 20.0 | putative homing endonuclease | GeneID:[80099627](https://www.ncbi.nlm.nih.gov/gene/80099627) |
| ORF97 | 56556 | 56894 | 112 | - | 12.9 | hypothetical protein | GeneID:[80099628](https://www.ncbi.nlm.nih.gov/gene/80099628) |
| ORF98 | 57099 | 58619 | 506 | + | 57.1 | putative terminase large subunit | GeneID:[80099629](https://www.ncbi.nlm.nih.gov/gene/80099629) |
| ORF99 | 58632 | 60071 | 479 | + | 54.3 | hypothetical protein | GeneID:[80099630](https://www.ncbi.nlm.nih.gov/gene/80099630) |
| ORF100 | 60081 | 60551 | 156 | + | 17.2 | putative methyltransferase | GeneID:[80099631](https://www.ncbi.nlm.nih.gov/gene/80099631) |
| ORF101 | 60548 | 61465 | 305 | + | 33.1 | hypothetical protein | GeneID:[80099632](https://www.ncbi.nlm.nih.gov/gene/80099632) |
| ORF102 | 61493 | 61903 | 136 | + | 14.9 | hypothetical protein | GeneID:[80099633](https://www.ncbi.nlm.nih.gov/gene/80099633) |
| ORF103 | 61947 | 62981 | 344 | + | 39.4 | major capsid protein | GeneID:[80099634](https://www.ncbi.nlm.nih.gov/gene/80099634) |
| ORF104 | 63034 | 63309 | 91 | + | 10.5 | hypothetical protein | GeneID:[80099635](https://www.ncbi.nlm.nih.gov/gene/80099635) |
| ORF105 | 63624 | 63896 | 90 | + | 10.5 | putative RNA polymerase | GeneID:[80099636](https://www.ncbi.nlm.nih.gov/gene/80099636) |
| ORF106 | 63896 | 64276 | 126 | + | 14.4 | hypothetical protein | GeneID:[80099637](https://www.ncbi.nlm.nih.gov/gene/80099637) |
| ORF107 | 64273 | 64836 | 187 | + | 21.3 | hypothetical protein | GeneID:[80099638](https://www.ncbi.nlm.nih.gov/gene/80099638) |
| ORF108 | 64849 | 66135 | 428 | + | 46.4 | hypothetical protein | GeneID:[80099639](https://www.ncbi.nlm.nih.gov/gene/80099639) |
| ORF109 | 66166 | 66690 | 174 | + | 19.0 | DUF3277 family protein | GeneID:[80099640](https://www.ncbi.nlm.nih.gov/gene/80099640) |
| ORF110 | 66894 | 67265 | 123 | + | 13.5 | DUF3277 domain-containing protein | GeneID:[80099641](https://www.ncbi.nlm.nih.gov/gene/80099641) |
| ORF111 | 67265 | 67744 | 159 | + | 17.7 | hypothetical protein | GeneID:[80099642](https://www.ncbi.nlm.nih.gov/gene/80099642) |
| ORF112 | 67758 | 68129 | 123 | + | 13.6 | hypothetical protein | GeneID:[80099643](https://www.ncbi.nlm.nih.gov/gene/80099643) |
| ORF113 | 68386 | 70752 | 788 | + | 86.0 | putative tape measure protein | GeneID:[80099644](https://www.ncbi.nlm.nih.gov/gene/80099644) |
| ORF114 | 70749 | 71510 | 253 | + | 28.5 | hypothetical protein | GeneID:[80099645](https://www.ncbi.nlm.nih.gov/gene/80099645) |
| ORF115 | 71516 | 71872 | 118 | + | 14.0 | hypothetical protein | GeneID:[80099646](https://www.ncbi.nlm.nih.gov/gene/80099646) |
| ORF116 | 72127 | 72786 | 219 | + | 24.2 | putative structural protein | GeneID:[80099647](https://www.ncbi.nlm.nih.gov/gene/80099647) |
| ORF117 | 72783 | 73523 | 246 | + | 26.7 | baseplate protein | GeneID:[80099648](https://www.ncbi.nlm.nih.gov/gene/80099648) |
| ORF118 | 73535 | 73906 | 123 | + | 14.2 | hypothetical protein | GeneID:[80099649](https://www.ncbi.nlm.nih.gov/gene/80099649) |
| ORF119 | 73908 | 75371 | 487 | + | 52.4 | baseplate-related protein | GeneID:[80099650](https://www.ncbi.nlm.nih.gov/gene/80099650) |
| ORF120 | 75390 | 76121 | 243 | + | 26.7 | hypothetical protein | GeneID:[80099651](https://www.ncbi.nlm.nih.gov/gene/80099651) |
| ORF121 | 76132 | 78189 | 685 | + | 71.7 | putative tail fiber protein | GeneID:[80099652](https://www.ncbi.nlm.nih.gov/gene/80099652) |
| ORF122 | 78233 | 78607 | 124 | + | 14.5 | hypothetical protein | GeneID:[80099653](https://www.ncbi.nlm.nih.gov/gene/80099653) |
| ORF123 | 78619 | 78972 | 117 | + | 13.0 | putative tail fiber protein | GeneID:[80099654](https://www.ncbi.nlm.nih.gov/gene/80099654) |
| ORF124 | 79032 | 80051 | 339 | + | 35.3 | putative tail fiber protein | GeneID:[80099655](https://www.ncbi.nlm.nih.gov/gene/80099655) |
| ORF125 | 80068 | 80628 | 186 | + | 20.9 | N-acetylmuramidase family protein | GeneID:[80099656](https://www.ncbi.nlm.nih.gov/gene/80099656) |
| ORF126 | 80646 | 80900 | 84 | + | 9.1 | hypothetical protein | GeneID:[80099657](https://www.ncbi.nlm.nih.gov/gene/80099657) |
| ORF127 | 80887 | 81330 | 147 | + | 15.9 | hypothetical protein | GeneID:[80099658](https://www.ncbi.nlm.nih.gov/gene/80099658) |
| ORF128 | 81462 | 81767 | 101 | + | 11.4 | hypothetical protein | GeneID:[80099659](https://www.ncbi.nlm.nih.gov/gene/80099659) |
| ORF129 | 81803 | 82117 | 104 | + | 12.3 | hypothetical protein | GeneID:[80099660](https://www.ncbi.nlm.nih.gov/gene/80099660) |
| ORF130 | 82165 | 82473 | 102 | - | 11.4 | hypothetical protein | GeneID:[80099661](https://www.ncbi.nlm.nih.gov/gene/80099661) |
| ORF131 | 82484 | 82804 | 106 | - | 12.2 | hypothetical protein | GeneID:[80099662](https://www.ncbi.nlm.nih.gov/gene/80099662) |
| ORF132 | 82767 | 83189 | 140 | + | 15.9 | hypothetical protein | GeneID:[80099663](https://www.ncbi.nlm.nih.gov/gene/80099663) |
| ORF133 | 82806 | 83681 | 291 | - | 33.3 | hypothetical protein | GeneID:[80099664](https://www.ncbi.nlm.nih.gov/gene/80099664) |
| ORF134 | 83779 | 84930 | 383 | - | 43.3 | hypothetical protein | GeneID:[80099665](https://www.ncbi.nlm.nih.gov/gene/80099665) |
| ORF135 | 84962 | 85195 | 77 | - | 8.6 | hypothetical protein | GeneID:[80099666](https://www.ncbi.nlm.nih.gov/gene/80099666) |
| ORF136 | 85233 | 85466 | 77 | - | 8.5 | hypothetical protein | GeneID:[80099667](https://www.ncbi.nlm.nih.gov/gene/80099667) |
| ORF137 | 86191 | 86592 | 133 | + | 15.8 | hypothetical protein | GeneID:[80099668](https://www.ncbi.nlm.nih.gov/gene/80099668) |
| ORF138 | 86615 | 86869 | 84 | + | 9.4 | hypothetical protein | GeneID:[80099669](https://www.ncbi.nlm.nih.gov/gene/80099669) |
| ORF139 | 86871 | 87260 | 129 | + | 14.8 | HAD-like domain protein | GeneID:[80099670](https://www.ncbi.nlm.nih.gov/gene/80099670) |
| ORF140 | 87257 | 87922 | 221 | + | 24.5 | hypothetical protein | GeneID:[80099671](https://www.ncbi.nlm.nih.gov/gene/80099671) |
| ORF141 | 88079 | 88381 | 100 | + | 11.9 | hypothetical protein | GeneID:[80099672](https://www.ncbi.nlm.nih.gov/gene/80099672) |
| ORF142 | 88496 | 88804 | 102 | + | 12.0 | hypothetical protein | GeneID:[80099673](https://www.ncbi.nlm.nih.gov/gene/80099673) |
| ORF143 | 89010 | 89459 | 149 | - | 16.2 | hypothetical protein | GeneID:[80099674](https://www.ncbi.nlm.nih.gov/gene/80099674) |
| ORF144 | 89719 | 91581 | 620 | + | 70.6 | DNA primase/helicase | GeneID:[80099675](https://www.ncbi.nlm.nih.gov/gene/80099675) |
| ORF145 | 91609 | 92370 | 253 | - | 28.5 | hypothetical protein | GeneID:[80099676](https://www.ncbi.nlm.nih.gov/gene/80099676) |

**Supplementary Table S3**: Details of tRNAs predicted in the Henu5 genome

| **tRNA No.** | **Location (bp)** | **Length (bp)** | **Amino acid** | **Anticodon** |
| --- | --- | --- | --- | --- |
| 1 | 53787-53859 | 73 | Gln | TTG |
| 2 | 53941-54017 | 77 | Trp | CCA |
| 3 | 54065-54139 | 75 | Arg | TCT |
| 4 | 54147-54223 | 77 | Lys | TTT |
| 5 | 54401-54490 | 90 | Ser | AGA |
| 6 | 54497-54581 | 85 | Leu | TAG |
| 7 | 54792-54867 | 76 | Ile | GAT |
| 8 | 54877-54955 | 79 | Asp | GTC |
| 9 | 54960-55046 | 87 | Tyr | GTA |
| 10 | 55050-55130 | 81 | Met | CAT |
| 11 | 55412-55488 | 77 | Cys | GCA |
| 12 | 55499-55574 | 76 | Asn | GTT |
| 13 | 55637-55714 | 78 | Pro | TGG |
| 14 | 55886-55961 | 76 | Gly | TCC |
| 15 | 56485-56561 | 77 | Phe | GAA |
| 16 | 56568-56643 | 76 | Glu | TTC |
| 17 | 56965-57039 | 75 | Thr | TGT |

**Supplementary Table S4:** Identified mutations in the genome of the R3 mutant using *P. aeruginosa* PAO1 as reference.

| Strain | Gene ID | Type of mutation | Genome  Position | Length | Mutation site | REF | ALT | Functional Annotations |
| --- | --- | --- | --- | --- | --- | --- | --- | --- |
| R3 | PA0727 | INDEL | 795008 | 1293 | 509 | AAC | - | Hypothetical protein |
| R3 | PA0727 | INDEL | 795431 | 1293 | 934 | A | - | Hypothetical protein |
| R3 | PA0727 | INDEL | 795443 | 1293 | 944 | CT | CGTGT | Hypothetical protein |
| R3 | PA0727 | INDEL | 795459 | 1293 | 960 | CG | CAG | Hypothetical protein |
| R3 | PA0727 | INDEL | 795471 | 1293 | 972 | G | - | Hypothetical protein |
| R3 | PA5040 | SNP | 5676046 | 2145 | 1813 | A | C | Type 4 fimbrial biogenesis outer membrane protein PilQ |
| R3 | PA5087 | SNP | 5725880 | 870 | 469 | C | G | Hypothetical protein |
| R3 | PA5087 | SNP | 5725881 | 870 | 468 | A | G | Hypothetical protein |
| R3 | PA5087 | SNP | 5725882 | 870 | 467 | A | G | Hypothetical protein |

**Supplementary Table S5:** Identified mutations in the genome of the R6 mutant using *P. aeruginosa* PAO1 as reference.

| **Strain** | **Gene ID** | **Type of mutation** | **Genome**  **Position** | **Length** | **Mutation site** | **REF** | **ALT** | **Functional Annotations** |
| --- | --- | --- | --- | --- | --- | --- | --- | --- |
| R6 | PA0727 | INDEL | 795008 | 1293 | 509 | AAC | - | Hypothetical protein |
| R6 | PA0727 | INDEL | 795431 | 1293 | 934 | A | - | Hypothetical protein |
| R6 | PA0727 | INDEL | 795443 | 1293 | 944 | CT | CGTGT | Hypothetical protein |
| R6 | PA0727 | INDEL | 795459 | 1293 | 960 | CG | CAG | Hypothetical protein |
| R6 | PA0727 | INDEL | 795471 | 1293 | 972 | G | - | Hypothetical protein |
| R6 | PA2023 | INDEL | 2215421 | 840 | 322 | C | - | UTP-glucose-1-phosphate uridylyltransferase |
| R6 | PA2178 | SNP | 2399732 | 609 | 549 | C | G | Hypothetical protein |
| R6 | PA2178 | SNP | 2399733 | 609 | 548 | C | G | Hypothetical protein |
| R6 | PA3802 | SNP | 4261924 | 1290 | 436 | A | G | Histidine--tRNA ligase |
| R6 | PA4547 | SNP | 5095672 | 1338 | 688 | A | C | Two-component response regulator PilR |
| R6 | PA5087 | SNP | 5725880 | 870 | 469 | C | G | Hypothetical protein |
| R6 | PA5087 | SNP | 5725881 | 870 | 468 | A | G | Hypothetical protein |
| R6 | PA5087 | SNP | 5725882 | 870 | 467 | A | G | Hypothetical protein |

**Supplementary Table S6:** Identified mutations in the genome of the R14 mutant using *P. aeruginosa* PAO1 as reference.

| **Strain** | **Gene ID** | **Type of mutation** | **Genome**  **Position** | **Length** | **Mutation site** | **REF** | **ALT** | **Functional Annotations** |
| --- | --- | --- | --- | --- | --- | --- | --- | --- |
| R14 | PA0727 | INDEL | 795008 | 1293 | 509 | AAC | - | Hypothetical protein |
| R14 | PA0727 | INDEL | 795431 | 1293 | 934 | A | - | Hypothetical protein |
| R14 | PA0727 | INDEL | 795443 | 1293 | 944 | CT | CGTGT | Hypothetical protein |
| R14 | PA0727 | INDEL | 795459 | 1293 | 960 | CG | CAG | Hypothetical protein |
| R14 | PA0727 | INDEL | 795471 | 1293 | 972 | G | - | Hypothetical protein |
| R14 | PA0192 | SNP | 219525 | 2373 | 354 | C | T | TonB-dependent receptor |
| R14 | PA1270 | SNP | 1380724 | 2040 | 1312 | C | A | Hypothetical protein |
| R14 | PA1270 | SNP | 1380729 | 2040 | 1317 | C | A | Hypothetical protein |
| R14 | PA1408 | SNP | 1531912 | 2424 | 1117 | C | A | Hypothetical protein |
| R14 | PA4402 | SNP | 4933452 | 1218 | 268 | G | C | Bifunctional ornithine acetyltransferase/N-acetylglutamate synthase |
| R14 | PA4402 | SNP | 4933454 | 1218 | 266 | T | C | Bifunctional ornithine acetyltransferase/N-acetylglutamate synthase |
| R14 | PA5040 | SNP | 5676046 | 2145 | 1813 | A | C | Type 4 fimbrial biogenesis outer membrane protein PilQ |
